# Chemo-mechanical failure mechanisms of the silicon anode in solid-state batteries

**DOI:** 10.1038/s41563-023-01792-x

**Published:** 2024-01-26

**Authors:** Hanyu Huo, Ming Jiang, Yang Bai, Shamail Ahmed, Kerstin Volz, Hannah Hartmann, Anja Henss, Chandra Veer Singh, Dierk Raabe, Jürgen Janek

**Affiliations:** 1 Institute of Physical Chemistry, Justus Liebig University Giessen, Giessen, Germany; 2 Center for Materials Research (ZfM), Justus Liebig University Giessen, Giessen, Germany; 3 Institute of Physical Science and Information Technology, Anhui University, Hefei, China; 4 Max-Planck-Institut für Eisenforschung, Düsseldorf, Germany; 5 Materials Science Centre and Faculty of Physics, Philipps University Marburg, Marburg, Germany; 6 Department of Materials Science and Engineering, University of Toronto, Toronto, Ontario Canada; 7 Present Address: Department of Materials, University of Oxford, Oxford, UK

**Keywords:** Batteries, Batteries

## Abstract

Silicon is a promising anode material due to its high theoretical specific capacity, low lithiation potential and low lithium dendrite risk. Yet, the electrochemical performance of silicon anodes in solid-state batteries is still poor (for example, low actual specific capacity and fast capacity decay), hindering practical applications. Here the chemo-mechanical failure mechanisms of composite Si/Li_6_PS_5_Cl and solid-electrolyte-free silicon anodes are revealed by combining structural and chemical characterizations with theoretical simulations. The growth of the solid electrolyte interphase at the Si|Li_6_PS_5_Cl interface causes severe resistance increase in composite anodes, explaining their fast capacity decay. Solid-electrolyte-free silicon anodes show sufficient ionic and electronic conductivities, enabling a high specific capacity. However, microscale void formation during delithiation causes larger mechanical stress at the two-dimensional interfaces of these anodes than in composite anodes. Understanding these chemo-mechanical failure mechanisms of different anode architectures and the role of interphase formation helps to provide guidelines for the design of improved electrode materials.

## Abstract (web summary)

Although silicon anodes are promising for solid-state batteries, they still suffer from poor electrochemical performance. Chemo-mechanical failure mechanisms of composite Si|Li_6_PS_5_Cl and solid-electrolyte-free silicon anodes are now revealed and should help in designing improved electrodes.

## Main

Solid-state batteries (SSBs) emerge as next-generation energy storage devices with high energy density and improved safety^1–3^. Compared with conventional batteries having liquid electrolytes, chemo-mechanics plays a more prominent role due to rigid solid/solid contacts and often have fairly different mechanical properties of the cell components^4,5^. Solid electrolytes (SEs) and active materials exhibit different chemical and mechanical properties, leading to complex chemo-mechanical interactions in SSBs, especially at the interfaces.

Silicon (Si), which plays a growing role as an anode component in lithium-ion batteries, has recently been explored as a promising alternative anode material in SSBs due to a similarly high theoretical capacity (3,590 mAh g^−1^ based on Li_3.75_Si at room temperature) compared with lithium metal^6,7^. The alloying process at a potential of *E* = 0.3 V (versus Li^+^/Li) avoids lithium metal nucleation and dendrite growth, as well as achieves higher energy density compared with other alloy anodes^8^. Moreover, the low cost and good stability of Si in air qualify it for large-scale manufacturing^9^. Due to large volume effects, Si anodes show Si particle pulverization and continuous solid electrolyte interphase (SEI) formation in liquid electrolytes, resulting in severe loss of lithium inventory^10^. In contrast, Si anodes in SSBs may show less or different SEI formation and particle pulverization due to the mechanical rigidity of inorganic SEs and external stack pressure, thus providing an opportunity to realize better cycling stability^11^.

However, strong volume changes in Si on lithiation/delithiation (that is, ~300% for the formation of Li_3.75_Si from Si) pose a challenge from the mechanics perspective and the underlying chemo-mechanical mechanisms remain elusive^12,13^. Three chemo-mechanical issues present particular challenges for the Si anodes in SSBs. (1) It is known that Si is not stable with sulfide SEs at low lithiation potential, leading to SEI formation at the Si|SE interface^14,15^. However, little work regarding the surface modification of Si particles (that is, coating layers) has been reported to date. Ion/electron percolation in composite Si anodes particularly suffers from these decomposition reactions. The SEI components, their microstructure and growth rate on cycling, therefore, require a better understanding. (2) Different from a composite anode with an interconnected three-dimensional (3D) interface, the use of a compact SE-free Si anode leads to a planar Si|SE interface (hereafter called two-dimensional (2D) interface and interphase for the sake of simplicity; Supplementary Fig. 1 and Supplementary Note 1), which causes less SEI degradation per mass of Si and reduces irreversible lithium loss^16–18^. However, pure Si is a semiconductor, and increasing the thickness of sputtered Si-film anodes to over 1 μm causes insufficient ion/electron transport^12^. The partial ionic/electronic conductivity of SE-free Si anodes lacks quantitative investigation so far, especially at different states of charge (SoC). Whether SE/conductive carbon additives or specific doping are necessary to support the ion/electron transport requires clarification. (3) Contact loss at Si|SE interfaces is less probable during the lithiation processes due to the volume expansion of Si, yet whether the interfaces remain stable during delithiation processes is an open question, especially for the 2D interface.

This work aims to better understand the interplay between lithium transport, microstructure evolution and the associated mechanical misfit effects across the heterointerfaces to reveal the failure mechanisms of both composite Si/Li_6_PS_5_Cl (LPSCl) and SE-free Si anodes in SSBs. The SEI components and the microstructure of the interfaces are investigated by combined multiscale (atomic scale to battery-cell scale) chemical and microstructural characterizations. A three-electrode battery setup is applied to quantitatively evaluate the SEI growth rate. SiO_*x*_ as a surface impurity of Si particles is found to be involved in SEI formation, causing complex degradation pathways. The analysis of different Li_*x*_Si alloy structures reveals sufficient ionic/electronic conductivity of SE-free Si anodes, suggesting that ionic/electronic additives are not required. The SE-free Si anodes, without dispersed electronically insulating components (that is, LPSCl and SEI), show even higher specific capacity than the Si/LPSCl composite anodes. However, scanning electron microscopy (SEM) investigations together with chemo-mechanical phase-field fracture models reveal a high maximum principal stress (−0.3 to 0.8 GPa) and increased plastic strain by 10% at the 2D Si|LPSCl interface, leading to 2 μm void formation at the 2D Si|LPSCl interface after the first delithiation and rapid capacity decay of SSBs based on SE-free Si anodes.

## (Electro)chemical stability of composite Si/LPSCl anodes

**Fig. 1:**
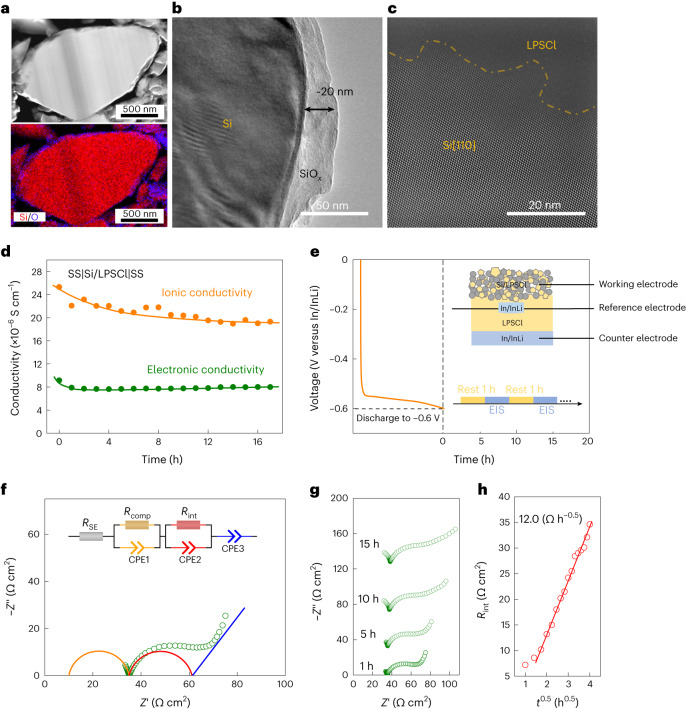
(Electro)chemical stability of composite Si/LPSCl anodes. **a**, HAADF-STEM image of Si particles and the corresponding EDS map. **b**, TEM image of a Si particle. **c**, Average-background-subtraction-filtered HAADF-STEM image at the Si|LPSCl interface. **d**, Electronic and ionic conductivities of the just-mixed Si/LPSCl as a function of time. **e**, Procedure for resting and impedance measurements based on a three-electrode cell. The inset shows the setup of the three-electrode cell. **f**, Nyquist plot and the corresponding equivalent circuit used to evaluate the impedance data (working electrode versus RE). **g**, Nyquist plots of a typical cell with long-term resting. **h**, *R*_int_ as a function of the square root of time (*t*^0.5^).

To evaluate the chemical stability of composite Si/LPSCl anodes, LPSCl with coarse particles (LPSCl (coarse)) was mixed with Si powder (Si/LPSCl weight ratio of 1/1, corresponding to a volume ratio of 0.68:1.00) in a mortar for 30 min. Supplementary Figs. 2 and 3 provide basic information about the Si and LPSCl used here. Oxygen is observed as an impurity at the surface of the Si particles, revealed by high-angle annular dark-field (HAADF) scanning transmission electron microscopy (STEM), showing SiO_*x*_ at the particle surface (Fig. 1a). The thickness of the SiO_*x*_ layer is ∼20 nm (Fig. 1b)^19^.

The average-background-subtraction-filtered STEM image (Fig. 1c) shows that no new crystalline phases have formed at the Si|LPSCl interface, indicating a chemically stable interface by direct contact, although amorphous interlayers cannot be totally excluded (Fig. 1c). We assume that SiO_*x*_ and LPSCl may chemically interact at the nanoscale, yet there is no clear evidence for reaction products forming before the lithiation of Si (Supplementary Fig. 4). To further investigate the stability of pristine Si/LPSCl composites and ion/electron transport across the composite, time-dependent electrochemical impedance spectroscopy (EIS) was applied based on a stainless steel SS|Si/LPSCl|SS cell setup (one measurement every hour) (Supplementary Fig. 5a). A transmission-line model was used to fit the impedances and split the ionic and electronic contributions in the mixed Si/LPSCl composite (Supplementary Fig. 5b and Supplementary Table 1)^20^. The ionic conductivity *σ*_ion_ of the composite Si/LPSCl decreases from 2.5 × 10^–5^ to 1.9 × 10^–5^ S cm^–1^ after 17 h, whereas the electronic conductivity *σ*_el_ remains relatively stable at *∼*8 × 10^–6^ S cm^–1^ after 17 h. The interfacial element diffusion shows more influence on the ionic conductivity than the electronic conductivity of the composite Si/LPSCl (Fig. 1d).

To study the electrochemical stability at the Si|LPSCl interface, In/InLi|LPSCl|Si/LPSCl cells were rested at the open-circuit voltage for ∼17 h after being discharged to *E* = –0.60 V, that is, to *E* = 0.02 V versus Li^+^/Li. Figure 1f illustrates the measurement procedure, where EIS measurements were conducted every hour. An In/InLi reference electrode (RE) was used to separate the cathode-related impedance (that is, the Si/LPSCl composite) from the total impedance of the whole cell^21^. Supplementary Fig. 6 shows the setup of the In/InLi RE. Figure 1f shows the initial EIS spectrum (working electrode versus RE) and the fitted equivalent circuit after discharging the battery to *E* = –0.6 V (versus In/InLi). A simplified model was used to fit the EIS spectra (Supplementary Note 2 and Supplementary Fig. 7). The impedance above ∼100 kHz corresponds to the ion transport (‘IR drop’) in the SE separator (*R*_SE_) and electrode composite (*R*_comp_), whereas the resistance in the range from ∼100 kHz to 0.5 Hz is mainly attributed to the contribution at the Si|LPSCl interface (*R*_int_) (ref. ^22^). The impedance plots depicted in Fig. 1g clearly show that *R*_int_ gradually increases during resting at the maximum lithium chemical potential, indicating interfacial instability. Also, *R*_int_ linearly increases with the square root of the resting time (*t*^0.5^), as described by a Wagner-type model for diffusion-controlled solid-state reactions (Fig. 1h and Supplementary Table 2)^23,24^. The slope *k*′, which corresponds to the SEI growth rate at the Si|LPSCl interface, was calculated to be *k*′ = 10.1 Ω h^−0.5^ at *T* = 25 °C and *p* = 50 MPa. Density functional theory (DFT) calculations show that the binding energy of Li/LPSCl is much larger than that of Li/Si (–7.21 eV versus –0.79 eV), indicating stronger reactivity between lithium and LPSCl (Supplementary Fig. 8). This simple comparison suggests that LPSCl is reduced by reaction with lithium from the Li_*x*_Si alloy at low potentials, leading to SEI formation and degradation at the Li_*x*_Si|LPSCl interface.

**Fig. 2:**
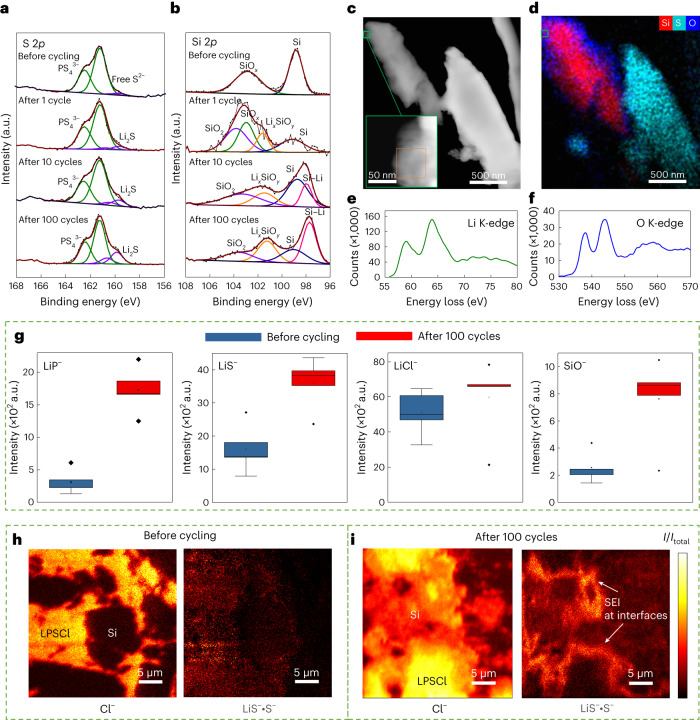
Characterization of SEI components at Si|LPSCl interfaces. **a**,**b**, S2*p* (**a**) and Si2*p* (**b**) XPS spectra of Si|LPSCl before cycling and after different cycles. **c**,**d**, HAADF cryo-STEM image (**c**) and corresponding EDS mapping (**d**) of Si/LPSCl after 100 cycles. **e**,**f**, Li K-edge (**e**) and O K-edge (**f**) EELS spectra of Si/LPSCl after 100 cycles. The orange box in the inset in **c** encloses the region from which EELS spectra were obtained. **g**, Box plots of SEI-related signal intensities (that is, LiP^–^, LiS^–^, LiCl^–^ and SiO^–^) from ToF-SIMS surface analyses of Si/LPSCl before and after 100 cycles. The SEI-related signal intensities were normalized by the S^–^ signal intensity. The lines in boxes depict the median and the lower- and upper-box limits indicate the 25th and 75th percentiles, respectively. The whiskers extend to ±1.5× interquartile range, and the points are outliers. **h**,**i**, ToF-SIMS images of the Cl^–^ fragment, and the product of the LiS^–^ and S^–^ fragments in Si|LPSCl composites before cycling (**h**) and after 100 cycles (**i**). All the signal intensities were normalized by the total signal intensity. HAADF cryo-STEM, HAADF-STEM operated under cryogenic conditions.

To investigate the electrochemical degradation products at the Li_*x*_Si|LPSCl interface, X-ray photoelectron spectroscopy (XPS) measurements were carried out before and after 1, 10 and 100 cycles of an In/InLi|LPSCl|Si/LPSCl cell. The strong double peaks at 161.7 eV and the very small double peaks at 160.1 eV in the S2*p* spectrum before cycling originate from the PS_4_^3−^ tetrahedra and ‘free’ S^2−^ ions of the argyrodite LPSCl structure, respectively (Fig. 2a)^25^. The increased intensity of the double peaks at 160.1 eV in the S2*p* spectrum occurs after one cycle, which corresponds to Li_2_S coming from the LPSCl decomposition with lithium. The intensity of Li_2_S further increases during the following cycles, indicating the continuous growth of the SEI. It should be noted here that the SiO_*x*_ impurity at the Si surface is also involved in SEI formation. The peaks at ∼98.7 and ∼102.9 eV in the Si2*p* spectrum before cycling originate from Si and SiO_*x*_, respectively (Fig. 2b)^17^. SiO_2_ (∼102.9 eV) and Li_*x*_SiO_*y*_ (∼101.4 eV) are observed after one cycle due to the reaction between SiO_*x*_ and lithium^26^. We assume that the disproportionation of SiO_*x*_ according to the reaction SiO_*x*_ = (*x*/2)SiO_2_ + (1 – *x*/2)Si is driven by the lithiation of the forming Si, and thus, SiO_2_ is formed apparently together with a Li_*x*_SiO_*y*_ phase. Figure 2c shows a HAADF cryo-STEM image of Si and LPSCl particles in contact with each other after 100 cycles. Figure 2d is the corresponding energy-dispersive X-ray spectroscopy (EDS) map showing the elemental distributions. The electron energy loss spectroscopy (EELS) K-edge structures of lithium match well with the simulated and experimental inelastic X-ray scattering spectra of lithium in Li_2_O (Fig. 2e)^27^. However, the prepeak and K-edge peak of O (Fig. 2f) are shifted to higher values (∼538 and ∼544 eV, respectively) compared with the corresponding simulated and experimental inelastic X-ray scattering data (∼535 and ∼541 eV, respectively)^27^. Additionally, the prepeak-to-K-edge peak intensity ratio of O (Fig. 2f) is slightly lower than the corresponding simulated and experimental ratios in the inelastic X-ray scattering data. The small differences in the O prepeak and K-edge structures may arise from the presence of SiO_2_ in Li_2_O. In addition, irreversible lithium after 10 cycles leads to the formation of a Li–Si peak at ∼98.0 eV in the Si2*p* spectrum (Fig. 2b). Note that the XPS signal of a small amount of Li–Si after one cycle may be buried by the SEI layer.

Time-of-flight secondary ion mass spectrometry (ToF-SIMS) was used to further confirm the local decomposition of the LPSCl electrolyte in the composite anode after cycling. The analysis of the composite surface after 100 cycles (Fig. 2g) revealed an increase in LiP^−^, LiS^−^ and LiCl^−^ signal intensities. These signals can be attributed to interfacial decomposition products of LPSCl (that is, Li_3_P, Li_2_S and LiCl)^25^. Additionally, an increase in the SiO^−^ signal intensity was observed after 100 cycles. The presence of Li^+^ ions can enhance the ionization of SiO_*x*_ fragments, resulting in a higher SiO^−^ signal intensity, which we consider to be a confirmation of the XPS results, suggesting the formation of SiO_2_ and Li_*x*_SiO_*y*_. Figure 2h,i shows the ToF-SIMS mass images of Si particles in the LPSCl matrix before cycling and after 100 cycles, respectively. In particular, after cycling, a layer with higher sulfur fragment intensity was found around the silicon particles. We interpret this as clear evidence for the Li_2_S-rich SEI at the Si|LPSCl interface. Summarizing the results obtained from different characterization techniques, electrochemical degradation occurs at the Si|LPSCl interface (including SiO_*x*_) and the components in the SEI layer most probably include the LPSCl decomposition products (that is, Li_3_P, Li_2_S and LiCl), as well as SiO_*x*_-derived SEI components (that is, SiO_2_, Li_*x*_SiO_*y*_ and Li_2_O).

## Ion/electron transport in SE-free Si anodes

**Fig. 3:**
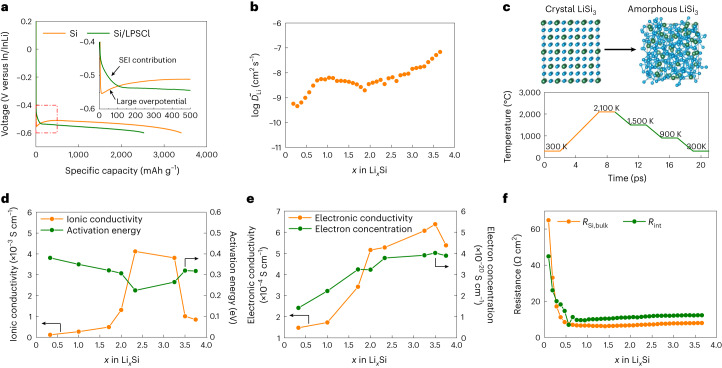
Ion/electron transport in SE-free Si anodes. **a**, Lithiation curves of the SE-free Si and Si/LPSCl anodes at 0.1C. **b**, Lithium chemical diffusion coefficient of the Li_*x*_Si alloys for different SoCs. **c**, Simulated crystal and amorphous LiSi_3_ structures before and after the melt-and-quench process. **d**, Simulated ionic conductivity and the corresponding activation energy of amorphous Li_*x*_Si alloys for different SoCs at 300 K. **e**, Simulated electronic conductivity and the corresponding electron concentration of amorphous Li_*x*_Si alloys for different SoCs at 300 K. **f**, Resistance changes in the Si anode during the lithiation process.

To avoid the detrimental effect of interfacial degradation in the composite Si/LPSCl anodes during cycling, SE-free Si anodes (that is, 99.5 wt% Si + 0.5 wt% polyvinylidene fluoride binder) were fabricated, which enable a 2D Si|LPSCl interface with less SEI formation per mass of Si. In/InLi|LPSCl|Si/LPSCl and In/InLi|LPSCl|Si cells were cycled at 0.1C to compare the specific capacity of Si/LPSCl composites and SE-free Si anodes. Although a large overpotential is observed during the initial lithiation process, the SE-free Si anode shows gradually decreased overpotential during the following lithiations, delivering a specific capacity of *q*_m_ ≈ 3,400 mAh g^–1^ (Fig. 3a). In contrast, the Si/LPSCl anode exhibits a comparably low specific capacity of *q*_m_ ≈ 2,600 mAh g^–1^, including the additional capacity (*q*_SEI_ ≈ 120 mAh g^–1^) from SEI formation. These results indicate that the SE-free Si anode exhibits sufficient mixed conductivity once a small amount of lithium is introduced. The optimization of the Si/LPSCl ratios in the composite Si anodes shows few effects on the improvement of specific capacity (Supplementary Fig. 9).

To quantify the ion/electron transport in the SE-free Si anode at different SoCs, the (apparent) lithium chemical diffusion coefficient $\widetilde{D}$_Li_ was measured by the galvanostatic intermittent titration technique (GITT)^28,29^. The measured $\widetilde{D}$_Li_(Li_*x*_Si) confirms the improved lithium diffusion kinetics with increased lithium concentration. We evaluate $\widetilde{D}$_Li_(Li_0.188_Si) = 5.7 × 10^–10^ cm^2^ s^–1^ at a low SoC, and the diffusion coefficient increased by two orders of magnitude to $\widetilde{D}$_Li_(Li_3.656_Si) = 6.9 × 10^–8^ cm^2^ s^–1^ in the fully lithiated state (Fig. 3b). The average $\widetilde{D}$_Li_ of SE-free Si anodes (1.0 × 10^–8^ cm^2^ s^–1^) is two orders of magnitude larger than that of LiNi_0.8_Co_0.1_Mn_0.1_O_2_ cathode materials reported in the literature^29^, indicating that ionic/electronic additives are not necessary for Si anodes.

The partial ionic/electronic conductivities of Li_*x*_Si during lithiation were calculated based on DFT simulations to support the high specific capacity of SE-free Si anodes. The crystal structures of Li_*x*_Si alloys were obtained from the Materials Project database, including LiSi_3_ (Li_0.33_Si), LiSi, Li_12_Si_7_ (Li_1.71_Si), Li_2_Si, Li_7_Si_3_ (Li_2.33_Si), Li_13_Si_4_ (Li_3.25_Si), Li_7_Si_2_ (Li_3.5_Si) and Li_15_Si_4_ (Li_3.75_Si) (Supplementary Table 3). Since the electrochemical lithiation process transforms the originally crystalline Si phases into amorphous Li_*x*_Si (refs. ^30,31^), a melt-and-quench process was applied to obtain amorphous structures for these Li_*x*_Si alloys (Fig. 3c). The radial distribution function of various Li_*x*_Si alloys clearly shows that no sharp second-neighbour peak is present, confirming the amorphous nature (lack of long-range order) of the Li_*x*_Si alloys (Supplementary Fig. 10).

The ionic conductivity of LiSi_3_ (Li_0.33_Si) is *σ*_ion_ = 1.2 × 10^–4^ S cm^–1^. The ionic conductivity further increases to 4.1 × 10^–3^ S cm^–1^ during lithiation from LiSi_3_ (Li_0.33_Si) to Li_7_Si_3_ (Li_2.33_Si), and then gradually decreases to 8.5 × 10^–4^ S cm^–1^ for the following lithiation to Li_15_Si_4_ (Li_3.75_Si) (Fig. 3d). The change in activation energy calculated from the temperature-dependent mean square displacement shows the opposite trend (Supplementary Fig. 11). The increasing Li^+^ concentration improves the ionic conductivity. At a low lithium concentration, the Li^+^ ions find enough vacant sites for fast diffusion^32^. Excessively high Li^+^ concentration leads to a lack of vacancies for Li^+^ migration, thereby decreasing the ionic diffusivity and conductivity of Li_*x*_Si (ref. ^33^). Therefore, a moderate Li^+^ concentration of Li_7_Si_3_ (Li_2.33_Si) (that is, 3.49 × 10^–20^ cm^3^) results in the highest ionic conductivity of *σ*_ion_ = 4.1 × 10^–3^ S cm^–1^ with the lowest activation energy of 0.225 eV (Supplementary Table 4). In parallel, a high electron concentration generally leads to a high electronic conductivity. Figure 3e shows the electronic conductivity *σ*_el_ increasing from 1.5 × 10^–4^ to 6.4 × 10^–4^ S cm^–1^ due to the increased electron concentration from LiSi_3_ (Li_0.33_Si) to Li_7_Si_2_ (Li_3.5_Si), respectively (Supplementary Table 4). Li_15_Si_4_ (Li_3.75_Si) shows a slightly decreased electron concentration and therefore a slightly lower electronic conductivity (that is, *σ*_el_ = 5.4 × 10^–4^ S cm^–1^). Note that the simulated ionic/electronic conductivity based on bulk structure features alone may differ from the experimental results, which are influenced by impurities, grain boundaries and other microstructural defects. Although the various Li_*x*_Si alloys have not yet been synthesized and studied with respect to their ionic/electronic conductivity, the obtained theoretical data are consistent with the experimental $\widetilde{D}$_Li_ values in the usual error ranges (Supplementary Note 3). In any case, the arithmetic averages of ionic conductivity (${\bar{\sigma }}_{{\rm{ion}}}$ = 1.5 × 10^–3^ S cm^–1^) and electronic conductivity (${\bar{\sigma }}_{{\rm{el}}}$ = 4.4 × 10^–4^ S cm^–1^) based on DFT simulations confirm the sufficient ionic/electronic conductivity of lithiated SE-free Si anodes without additional additives, which enables good rate performance (Supplementary Fig. 12).

Impedance measurements were conducted during every voltage relaxation of the GITT measurement to evaluate Li^+^ transport across the 2D Si|LPSCl interface (Supplementary Fig. 13 and Supplementary Table 5). Although the ionic/electronic conductivity continuously changes during relaxation, the resistance of the Si bulk (*R*_Si,bulk_) first decreases from 64.9 to 6.7 Ω cm^2^ after lithiation to Li_0.75_Si and then remains at a low value (∼7.0 Ω cm^2^) for the following lithiation (Fig. 3f). The low *R*_Si,bulk_ is consistent with the high ionic/electronic conductivity and enables the fast lithium transport in the SE-free Si anode (Fig. 3f). The interface resistance *R*_int_ decreases from 44.9 to 9.7 Ω cm^2^ after lithiation to Li_0.75_Si due to the improved interface dynamics and then slightly increases to 12.3 Ω cm^2^ after full lithiation due to 2D SEI formation. In addition, the time-dependent impedances were measured during resting at the open-circuit voltage after full lithiation. The calculated rate constant is *k*′ = 0.3 Ω h^−0.5^, which is much lower than that for the Si/LPSCl anode (that is, *k*′ = 10.1 Ω h^−0.5^) (Supplementary Fig. 14 and Supplementary Table 6). Assuming that the SEI growth kinetics does not depend on the geometry of the Si surface, this result indicates that the SEI growth at the 3D Si|LPSCl interface strongly contributes to a higher tortuosity in the Si/LPSCl composite.

## Cycling stability at the 2D and 3D Si|LPSCl interfaces

**Fig. 4:**
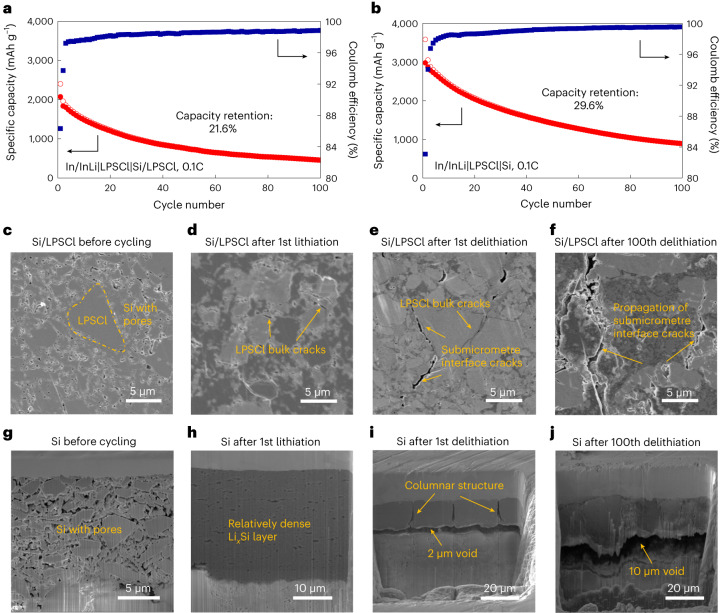
Cycling stability at the 2D and 3D Si|LPSCl interfaces. **a**,**b**, Cycling performance of the In/InLi|LPSCl|Si/LPSCl cell (**a**) and the In/InLi|LPSCl|Si cell (**b**) at 0.1C under 50 MPa. **c**–**f**, Cross-sectional SEM images of the Si/LPSCl anode before cycling (**c**), after the first lithiation (**d**), after the first delithiation (**e**) and after the 100th delithiation (**f**). **g**–**j**, Cross-sectional SEM images of the SE-free Si anode before cycling (**g**), after the first lithiation (**h**), after the first delithiation (**i**) and after the 100th delithiation (**j**).

The In/InLi|LPSCl|Si/LPSCl and In/InLi|LPSCl|Si cells were cycled at 0.1C under 50 MPa to investigate the long-term cycling stability. The capacity retention of the In/LiIn|LPSCl|Si/LPSCl cell is 21.9% after 100 cycles (Fig. 4a). The poor cycling stability comes from the continuous 3D SEI growth in the composite Si/LPSCl, which has been revealed in the discussion above (Fig. 2). The electronically conducting vapour-grown carbon fibre (VGCF) additive leads to even more severe SEI degradation, as the carbon has the same potential as Si, which causes worse cycling stability for the composite Si/LPSCl/VGCF anodes (Supplementary Fig. 15). SE-free Si anodes with their simple layer geometry show less interface degradation per volume of SE and the Li^+^ ions only need to pass the one 2D SEI, which causes less overpotential at the anode^16–18^. However, the capacity retention of the In/LiIn|LPSCl|Si cell is only 29.3% after 100 cycles (Fig. 4b) under the given experimental conditions. To understand the poor cycling stability of the SE-free Si anodes, the microstructure evolution of Si/LPSCl composites and SE-free Si anodes are compared in the following.

LPSCl particles and porous regions of aggregated Si particles are observed for the Si/LPSCl anode before cycling (Fig. 4c). These regions of Si particles with a high hardness of ∼10.6 GPa cannot be densified at 380 MPa during fabrication^34^, whereas the relatively soft LPSCl enables close contact with Si particles after pressing under 380 MPa. Lithium incorporation into Si shows an elastic softening effect, which decreases the hardness of fully lithiated Li_3.75_Si to 1.5 GPa (refs. ^35,36^). Si expansion under a constraining pressure of 50 MPa tends to densify the microstructure of relatively soft Li_*x*_Si, leading to an interconnected Li_*x*_Si microstructure after the first lithiation (Fig. 4d). Although the 3D Si|LPSCl interfaces remain intact, the stress generated by Si expansion causes crack formation inside the bulk LPSCl particles (Fig. 4d and Supplementary Fig. 16a). Submicrometre cracks are observed at the 3D Si|LPSCl interfaces after the first delithiation due to the shrinkage of Si (Fig. 4e). These submicrometre cracks propagate and widen after the 10th and 100th delithiation (Supplementary Fig. 16b and Fig. 4f). We note that the LPSCl|Si/LPSCl interface maintains close contact even after 100 cycles (Supplementary Fig. 16c).

The SE-free Si anode shows discrete Si particles with many voids before cycling due to the shape and hardness of Si particles (Fig. 4g). The thickness is 11.5 μm and the porosity is calculated to be 40.4% based on the theoretical density of 2.4 g cm^−3^ (refs. ^17,34^). The Si expansion not only assures good 2D Si|LPSCl interface contact but also densifies the Si layer. A dense and interconnected Li_*x*_Si microstructure with much less porosity is observed after the first lithiation, where most voids have vanished (Fig. 4h). The Si layer shows a columnar microstructure after the first delithiation (Fig. 4i and Supplementary Fig. 17a). It does not revert to the original homogeneous microstructure on delithiation, indicating irreversible plastic deformation. Different from the submicrometre cracks at the interfaces of the 3D Si/LPSCl composite, a 2-μm-wide void is observed at the 2D Si|LPSCl interface (Fig. 4i). An increased void thickness of 10 μm is observed after the 100th delithiation, indicating serious aging and mechanical degradation of the 2D Si|LPSCl interface during repeated cycling (Supplementary Fig. 17b and Fig. 4j). The EIS spectra reflect this by the increased *R*_int_, further confirming that the contact loss causes a poor cycling stability of SE-free Si anodes (Supplementary Fig. 18 and Supplementary Table 7). Note that the formed SEI layer is also unstable, leading to contact loss from the SE-free Si anode after the first delithiation (Supplementary Fig. 19). A fully coupled chemo-mechanical phase-field fracture model was developed to evaluate the stress and void formation at 2D and 3D interfaces during the first lithiation and delithiation processes (Supplementary Note 4 and Supplementary Figs. 20 and 21).

## SE-free Si anodes in Si|LPSCl|NCM@LBO full cells

**Fig. 5:**
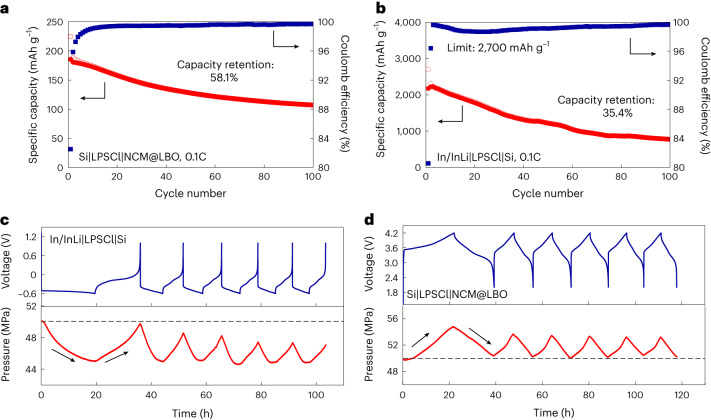
SE-free Si anodes in Si|LPSCl|NCM@LBO full cells. **a**,**b**, Cycling performance of the Si|LPSCl|NCM@LBO full cell (**a**) and the In/InLi|LPSCl|Si half-cell (**b**) with a cut specific capacity of 2,700 mAh g^–1^ at 0.1C. **c**,**d**, Galvanostatic cycling of the In/InLi|LPSCl|Si half-cell (**c**) and the Si|LPSCl|NCM@LBO full cell (**d**) along with the measured stack-pressure changes. Each dataset shows six cycles that were performed at 0.05C for the first cycle and 0.10C for the following cycles. The pressure at *t* = 0 is 50 MPa.

Composite LiNi_0.83_Co_0.11_Mn_0.06_O_2_ (NCM) cathodes were paired with SE-free Si sheet anodes with an N/P ratio of 1.3 in full cells. The surfaces of the NCM particles were coated with a thin layer (2 nm) of Li_2_B_4_O_7_ (LBO) to prevent electrochemical degradation at the NCM|LPSCl interface. The areal capacity of the NCM cathode was *q*_a_ = 4.31 mAh cm^–2^, which corresponds to a thickness of ∼125 μm (Supplementary Fig. 22a). We observed a substantial influence of the microstructure on the performance of the relatively thick NCM cathodes. NCM cathodes with a small particle size of LPSCl (NCM@LBO (small)) show better performance than those with coarse LPSCl (NCM@LBO (coarse)) due to the improved homogeneity of the microstructure (Supplementary Fig. 22). Although SE pellets fabricated by LPSCl (small) show a lower ionic conductivity than those prepared by LPSCl (coarse), a homogeneous distribution of NCM particles in the LPSCl matrix is observed in NCM@LBO (small), which is beneficial for fast ion/electron transport (Supplementary Figs. 22 and 23). The Si|LPSCl|NCM@LBO (small) cells deliver an initial specific discharge capacity of 185.6 mAh g^–1^ and can successfully operate 100 cycles with a capacity retention of 58.1% (Fig. 5a).

Full SSB cells normally exhibit worse cycling performance compared with half-cells with a Li metal anode due to the fast loss of the lithium balance—driven by irreversible capacities in both anode and cathode. The Si|LPSCl|NCM@LBO full cells show better capacity retention compared with the In/InLi|LPSCl|Si half-cells after 100 cycles at 0.1C (58.1% versus 29.6%). To rationalize this, we first checked whether this comes from incomplete lithiation in full cells due to the N/P ratio of 1.3 (ref. ^37^). The In/InLi|LPSCl|Si cells were cycled at 0.1C with a cutoff at a specific capacity of 2,700 mAh g^–1^ (which is the same degree of lithiation in full cells) for comparison. The capacity retention was 35.4% after 100 cycles, indicating that the improved cycling stability of full cells does not primarily come from partial lithiation (Fig. 5b). In the next step, the stack-pressure evolution was investigated during galvanostatic cycling. The pristine pressure was 50 MPa. The In/InLi|LPSCl|Si cell shows a negative pressure change (that is, *p* < 50 MPa) during cycling, indicating that the volume change in In/InLi is actually larger than that in the SE-free Si sheet electrode (Fig. 5c). We speculate that the positive pressure change (that is, *p* > 50 MPa) of the Si|LPSCl|NCM@LBO cell helps to maintain the cycling stability of full cells (Fig. 5d). In addition, the Si/LPSCl composite anode in a Si/LPSCl|LPSCl|NCM@LBO cell also shows a positive pressure change (Supplementary Fig. 24). We note that half-cells may not be a good choice for the preliminary evaluation of new electrodes under confined pressure conditions, as chemo-mechanics can seriously interfere and lead to different behaviours of full cells.

The contact loss at the 2D Si|LPSCl interface was also observed in full cells after 100 cycles (Supplementary Fig. 25a). A polypropylene carbonate (PPC) layer with a thickness of ∼1 μm was coated as a mechanical buffer layer on the surface of Si sheet anodes (Supplementary Fig. 25b). The PPC layer not only blocks interface degradation but also alleviates interface stress and helps to maintain contact. The Si@PPC|LPSCl|NCM@LBO cell shows a capacity retention of 71.9% after 100 cycles (Supplementary Fig. 25c). Close contact without voids is observed at the 2D Si|LPSCl interface, confirming the effectiveness of the interface modification by a PPC layer (Supplementary Fig. 25d). However, the initial specific discharge capacity of the NCM cathodes decreases from ∼180 to ∼160 mAh g^–1^, which may come from unsatisfactory ion transport across the PPC-related interfaces (that is, LPSCl|PPC and PPC|Si interfaces). Therefore, better modification layers with both high ionic conductivity and good compatibility with LPSCl/Si are required.

In summary, we explored the chemo-mechanical failure mechanisms of both Si/LPSCl composite and SE-free Si anodes using a correlative approach with several experimental and simulation methods. The following key results are obtained. (1) SEI growth kinetics and compositions: the impedance analysis of three-electrode cells shows that the rate constant *k*′ describing the resistance increase due to SEI growth is much larger for the Si/LPSCl composite anodes compared with SE-free Si anodes (10.1 versus 0.3 Ω h^−0.5^). The components of the SEI include the LPSCl decomposition products (that is, Li_3_P, Li_2_S and LiCl). The SiO_*x*_ surface layer on the Si particles disproprotionates during lithiation into Si and SiO_2_, and related phases enter into the SEI (that is, SiO_2_, Li_2_O and Li_*x*_SiO_*y*_). We note that the SE-free Si anode with a planar interface offers valuable information about SEI formation and its kinetics. SEI formation in 3D Si composites can be highly detrimental to cell capacity, particularly for high volume fractions of Si. (2) Lithiation/delithiation kinetics: the SE-free Si anodes show an average $\widetilde{D}$_Li_ = 1.0 × 10^–8^ cm^2^ s^–1^ during lithiation (obtained from GITT). DFT simulations deliver an average ionic conductivity and electronic conductivity of *σ*_ion_ = 1.5 × 10^–3^ S cm^–1^ and *σ*_el_ = 4.4 × 10^–4^ S cm^–1^, respectively, enabling a low *R*_Si,bulk_ ≈ 7 Ω cm^2^ for fast ion/electron transport. The obtained theoretical and experimental data for the partial conductivities, thermodynamic factor and chemical diffusion coefficient are consistent within usual error ranges, which gives strong support for our approach. The SE-free Si anodes show even higher specific capacity than Si/LPSCl composite anodes (∼3,400 versus ∼2,600 mAh g^–1^) due to the unhindered interface by electronically insulative components (that is, LPSCl and SEI). (3) Chemo-mechanics of Si anodes: the 2D Si|LPSCl interface of the SE-free Si anodes shows similarly poor cycling stability compared with 3D Si|LPSCl interfaces of the Si/LPSCl composite anodes. The ‘quasi-2D’ Si|LPSCl interface forms voids more readily compared with 3D interfaces. Chemo-mechanically coupled phase-field fracture modelling (Supplementary Note 4) reveals that a large stress (0.3 GPa) is accumulated at the 2D Si|LPSCl interface during the lithiation process, leading to ∼10% plastic strain of the LPSCl separator. A thin PPC modification layer can not only suppress interface degradation but also alleviate interface stresses, thereby maintaining good contact. We also highlight that half-cells using In/InLi anodes may not be a good choice for the preliminary evaluation of new electrodes due to the different behaviours of chemo-mechanics compared with full cells.

In view of all the results, we conclude that Si anodes provide a promising alternative to lithium metal anodes. The projected specific energy and energy density of Si-based SSBs are 300 Wh kg^–1^ and 800 Wh L^–1^, respectively, which are comparable with SSBs based on Li metal anodes. Our work provides a deep understanding of the role of SEI growth and the chemo-mechanics at 2D and 3D Li_*x*_Si|LPSCl interfaces on cell kinetics and capacity fading of SSBs, which helps to further improve Si anodes for use in SSBs. Future research should focus on improving the cycling stability and decrease the stack pressure. We are confident that commercialized Si-based SSBs with a high energy density will be developed in the future.

## Methods

### Materials preparation

Coarse-grained LPSCl particles were obtained from NEI Corporation and used as received for SEs, whereas small-grained LPSCl particles were obtained from Posco JK Solid Solution and used as received for composite cathodes. The Si particles (μ-Si, 1–5 μm, 99.9% metal basis purity) were obtained from Alfa Aesar and dried in a Büchi furnace at 80 °C overnight before use. Polyvinylidene fluoride binder used for the Si sheets was obtained from Kynar (HSV-900) and used as received. PPC (*M*_w_ = 50,000), lithium bis(trifluoromethanesulfonyl)imide (99.95%) and anhydrous acetonitrile were obtained from Sigma-Aldrich and used as received for the modification layer. VGCF (Sigma-Aldrich, iron free) has an average specific surface area of 24 m^2^ g^–1^ with a diameter of 100 nm and a fibre length of 20–200 μm. NCM cathode material with a surface coating of LBO (NCM@LBO) was obtained from MSE Supplies. NCM@LBO and VGCF were dried in a Büchi furnace at 200 °C overnight before use. An indium foil (Alfa Aesar, 99.99%, 100 μm thickness) and a lithium foil (Albemarle, Rockwood Lithium, 99.9%, 100 μm thickness) were used as received for the In/InLi alloy anodes.

### Preparation of different Si electrodes

To fabricate the SE-free Si sheet anodes, a slurry was prepared using 99.5 wt% Si particles, 0.5 wt% polyvinylidene fluoride binder and *N*-methyl-2-pyrrolidone solvent before casting on a copper current collector using a doctor blade. The cast sheet was dried under a vacuum at 80 °C overnight to remove the solvent followed by punching out the electrode discs (*∅* = 10 mm). The loading of Si in the SE-free Si sheet anodes is ∼1.6 mg cm^–2^ with a thickness of 11.5 μm. To fabricate the Si@PPC anodes, the PPC solution was first prepared. PPC (3.0 g) and lithium bis(trifluoromethanesulfonyl)imide (0.5 g) were added into anhydrous acetonitrile (10 ml) under intense stirring to form a homogeneous solution. Then, the PPC solution was blade cast on top of the Si sheet followed by drying under a vacuum at 80 °C overnight to remove the solvent. The Si@PPC sheet was punched to obtain the electrode discs (*∅* = 10 mm). The thickness of the PPC layer was ∼1 μm. To fabricate the Si/LPSCl composite anode in pressed pellets, Si and LPSCl (weight ratio = 1:1) were ground in a mortar for 30 min. Then, 4 mg Si/LPSCl composite powder was used and pressed together with LPSCl SE as the separator layer at 380 MPa. Note that 4 mg is the smallest mass of anode composite that could homogeneously cover the surface of the LPSCl SE separator (*∅* = 10 mm). The loading of Si in the Si/LPSCl anodes results as *m*_A_(Si) = 2.55 mg cm^–2^.

### Materials characterization

Crystal structures of samples were examined by X-ray diffraction using an Empyrean diffractometer (PANalytical) using Cu Kα radiation with 2*θ* in the range from 10.00° to 80.00° and a step size of 0.02°. The particle size distribution was measured by a particle size analyser (HELOS). The LPSCl and Si particles were distributed in xylene and distilled water, respectively. The surface morphology of the samples was investigated by a Merlin high-resolution SEM instrument (Carl Zeiss). The cross sections in this work were created and analysed using a TESCAN XEIA3 system equipped with a Xe-plasma focused-ion-beam (FIB) column and an EDAX Octane Elite EDS detector. A Leica EM VCT500 cryo-stage was used to avoid beam damage. XPS measurements were used to investigate the electrochemical degradation of Si/LPSCl samples. Measurements were carried out using a PHI5000 Versa Probe II instrument. All the samples were transferred to the instrument in an argon-filled transfer vessel. Monochromatic Al Kα radiation (1,486.6 eV) was used; the power of the X-ray source was 100 W, and the beam voltage was 20 kV. The examined areas were 1 mm^2^. All the data were calibrated to the signal of adventitious carbon at 284.8 eV.

Transmission electron microscopy (TEM) characterization was done using a double Cs-corrected JEOL 2200FS microscope operating at 200 kV. The Si samples shown in Fig. 1a and the Si/LPSCl samples shown in Fig. 1c and Supplementary Fig. 3 were first pressed into pellets at 380 MPa followed by FIB cutting. The transfer between FIB and TEM was optimized to minimize air exposure (that is, <10 s). The Si samples (Fig. 1b) and cycled Si/LPSCl samples (Fig. 2c–f) were prepared in an argon-filled glovebox and transferred to the TEM in an argon atmosphere using a double-tilt LN2 Atmos Defend Holder from Melbuild to completely avoid air exposure. To prepare the Si samples shown in Fig. 1b, the Si particles were poured over a carbon-film-coated Cu mesh grid in a glovebox, and the grids were then loaded into the double-tilt LN2 Atmos Defend Holder. To prepare the cycled Si/LPSCl samples (Fig. 2c–f), a pair of sharp tweezers was used to scratch some particles from the Si/LPSCl surface and deposit them on a carbon-film-coated Cu mesh grid. The grids were then transferred to the double-tilt LN2 Atmos Defend Holder. All the samples were investigated at room temperature, except for the cycled samples (Fig. 2c–f), which were measured at about −165 °C to minimize beam damage.

For ToF-SIMS, an M6 Hybrid SIMS instrument (IONTOF) was used. To compare the fragment intensity ratios, surface measurements in the spectrometry mode (high signal intensities and mass resolution; full-width at half-maximum *m*/Δ*m* = 3,374@*m*/*z* = 31.98 (S^−^)) were performed. The surfaces were not sputter cleaned before the measurements. Using Bi_3_^+^ ions with an energy of 30 keV as the primary ion species, 100 × 100 μm^2^ were analysed with 128 × 128 pixels^2^ in the sawtooth raster mode. After reaching a primary ion dose of 10^12^ ions cm^–2^, the measurements were stopped to achieve comparable measuring conditions. Five mass spectra each were recorded in the negative- and positive-ion mode at different locations on the surface. The measurements on the FIB crater walls were performed in the imaging mode (high lateral resolution; full-width at half-maximum *m*/Δ*m* = 97@*m*/*z* = 31.97 (S^−^)). The measured area was cleaned with the primary ion beam in long pulses (10%) for 2 min (pristine) or 4 min (cycled). Bi^+^ ions with an energy of 30 keV were used as the primary ion species. Areas between 45 × 45 μm^2^ and 75 × 75 μm^2^ were analysed with 1,024 × 1,024 pixels^2^ in the sawtooth raster mode. Since a high lateral resolution leads to a poor mass resolution, the signals of several fragments coincide into one broad signal. The intensities of LiX^−^ (X = P, S or Cl) fragments are, therefore, multiplied by the intensity of the X^−^ fragment to obtain images exclusively corresponding to LiX^−^ without the other adjacent fragments. The samples were electrically isolated from the sample holder by using a non-conductive tape and measured with the electron neutralization of the flood gun. Data evaluation was carried out with the SurfaceLab v.7.3 software (IONTOF).

### Electrochemical performance tests

Three-electrode cells were built with the same cell case and a two-part polyether ether ketone cylinder. To keep the separator intact during processing, 50 mg LPSCl was put into the polyether ether ketone cylinder and pressed into a pellet with a hand press. A 0.8 mg indium foil rolled on a thin stainless steel wire was put on the surface of the LPSCl pellet and served as the RE after lithiation. Another 50 mg LPSCl was added on top of the RE and pressed to form a separator from a total of 100 mg LPSCl with about 650 µm thickness. The anode and cathode in the three-electrode cells were the same as those in two-electrode cells. The impedance spectra were measured by a Biologic SP300 potentiostat, which were operated using a proprietary software (EC-Lab, BioLogic). The amplitude of the input signal was 10 mV, and the frequency range was from 1 MHz to 0.1 Hz. For the measurement of ionic conductivity, the sample powder was put in a cylindrical cell casing (*∅* = 12 mm). A pressure of 380 MPa was applied to compress the powder followed by a constant 50 MPa pressure (CompreDrive, rhd instruments) during the impedance measurements. Direct-current polarization was carried out for the measurement of electronic conductivity. The applied voltages were 0.5, 1.0, 1.5 and 2.0 V, with an equilibration time of 1 h at each voltage.

To quantitatively evaluate the SEI growth, a Wagner-type model for diffusion-controlled solid-state reactions was applied to describe the growth rate of the SEI layer^23,24^. The analysis relies on the assumption that charge transport across the SEI layer is mainly dominated by ions (that is, ${\sigma }_{{{\rm{Li}}}^{+}}$ ≫ ${\sigma }_{{\rm{e}}^{-}}$)^38^.

1 $${R}_{\mathrm{int}}=\frac{1}{S{\bar{\sigma }}_{\mathrm{int}}}\sqrt{\frac{{V}_{{\rm{m}}}}{x{F}^{2}}{\rm{\times }}\frac{{\bar{\sigma }}_{{{\rm{Li}}}^{+}}\times {\bar{\sigma }}_{{e}^{-}}}{{\bar{\sigma }}_{{{\rm{Li}}}^{+}}+{\bar{\sigma }}_{{e}^{-}}}{\rm{\times }}\Delta {{\rm{\mu }}}_{{\rm{Li}}}}{\rm{\times }}\sqrt{t}=\frac{1}{S{\bar{\sigma }}_{\mathrm{int}}}{\rm{\times }}k\sqrt{t}={k}^{{\prime} }\sqrt{t}\,$$

Here *S*, *F*, *x* and *t* denote the contact area, Faraday’s constant, number of moles of Li extracted from LPSCl and resting time, respectively. The average ionic conductivity of the SEI layer is denoted as ${\bar{\sigma }}_{\mathrm{int}}$. Also, *V*_m_ represents the average molar volume of the SEI. Furthermore, ${\bar{\sigma }}_{{{\rm{Li}}}^{+}}$ and ${\overline{\sigma }}_{{{\rm{e}}}^{\,-}}$ denote the mean partial ionic and electronic conductivities of the SEI layer, respectively. The difference in the lithium chemical potential across the SEI, Δ*µ*_Li_, which serves as the driving force for the SEI growth, is also included in the rate constant. The rate constants *k* and *k*′ reflect the growth rate in terms of thickness and resistance, respectively.

The GITT was applied to evaluate the lithium chemical diffusion coefficient $\widetilde{D}$_Li_ of the SE-free Si anode (Supplementary Fig. 26a)^28,29^. A short polarization at 0.1C (0.56 mA cm^–2^) for 15 min followed by a voltage relaxation for 2 h was carried out to measure its evolution at small intervals of SoC (that is, Δ*x* = 0.094 in the Li_*x*_Si alloy)^29^. This procedure allows the assumption of semi-infinite conditions^39^. The relaxation potential *U*_*i*_ was assumed to evolve with time according to equation (2):

2 $${U}_{i}\left(t\right)={U}_{0}-\frac{2}{\sqrt{\uppi }}I{Z}_{{\rm{W}}}({x}_{i})\sqrt{t}+{Ct},\,{\rm{where}}\,{Z}_{{\rm{W}}}({x}_{i})=\frac{W\left({x}_{i}\right){RT}}{{F}^{2}{Am}{c}_{0}\sqrt{{\widetilde{D}}_{{\rm{Li}}}}}.$$

The Warburg coefficient *Z*_W_ was obtained by fitting the *U*_*i*_(*t*) curve from 15 to 300 s (Supplementary Fig. 26b). The thermodynamic factor, $W=\frac{\partial \,\mathrm{ln}({a}_{{\rm{Li}}})}{\partial \,\mathrm{ln}({c}_{{\rm{Li}}})}=-\frac{F}{RT}x\frac{\partial U}{\partial x}$, is calculated from the *U*_0_ versus *c*_Li_ data recorded during the experiments (Supplementary Fig. 26c)^40^. *I*, *F* and *A* represent the polarization current, Faraday constant and Si|LPSCl interface area, respectively; *c*_0_ and *m* are the mass and lithium concentration of fully lithiated Si (that is, Li_3.75_Si), respectively.

All the cells were assembled in an argon-filled glovebox (*p*(O_2_)/*p* < 0.1 ppm and *p*(H_2_O)/*p* < 0.1 ppm; MBRAUN LABmaster SP). For assembling, a home-made pellet-type cell case with 10-mm-diameter polyether ether ketone sleeve and two stainless steel stamps were used. For InLi|LPSCl|Si half-cells, 80 mg LPSCl was first pressed by hand. A Si sheet anode (∼1.6 mg cm^–2^) was added on one side of the separator. Afterwards, the stacked pellet was pressed under 3 tons (∼380 MPa) for 3 min. An indium foil (*∅* = 9 mm, 100 μm thickness) and a lithium foil (*∅* = 8 mm, 100 μm thickness) were added on the other side of the separator to form the Li–In anode. In/InLi|LPSCl|Si/LPSCl half-cells were assembled in a similar way by using Si/LPSCl composites. The cells were fixed by a stainless steel frame to maintain a constant pressure (that is, 50 MPa). Galvanostatic cycling of the cells was carried out in the voltage range from −0.6 to 1.0 V.

The composite cathodes were prepared from NCM@LBO, LPSCl (small- or coarse-grained particles) and VGCF with a mass ratio of 80:20:3. To achieve uniform composite cathodes, the mixture was hand ground with an agate mortar for 30 min. The N/P ratio is 1.3 for the Si|LPSCl|NCM@LBO full cells, which is defined based on the theoretical capacities *q*_th_(Si) = 3,500 mAh g^−1^ and *q*_th_(NCM@LBO) = 200 mAh g^−1^. The InLi|LPSCl|NCM@LBO cells with the same NCM@LBO loading were assembled for comparison. Long-term charge and discharge tests were performed using a MACCOR battery cycler. Galvanostatic cycling of the cells was carried out in the voltage range from 2.0 to 4.2 V. Pressure change during cycling was tracked by a CompreDrive device (rhd instruments).

## Online content

Any methods, additional references, Nature Portfolio reporting summaries, source data, extended data, supplementary information, acknowledgements, peer review information; details of author contributions and competing interests; and statements of data and code availability are available at 10.1038/s41563-023-01792-x.

### Supplementary information

Supplementary Information Supplementary Notes 1–4, Figs. 1–26, Tables 1–8 and refs. 1–23.

## Data Availability

All the data generated and analysed in this study are included in the Article and its Supplementary Information. The data that support the plots within this paper are available via Zenodo at 10.5281/zenodo.10356036.
