# Supplementary material for: Chemo-mechanical failure mechanisms of the silicon anode in solid-state batteries
Source: Nat Mater. 2024 Jan 26;23(4):543–51. doi: 10.1038/s41563-023-01792-x (PMC10990934; doi:10.1038/s41563-023-01792-x)
Supplement: Supplementary file 1 — Supplementary Notes 1–4, Figs. 1–26, Tables 1–8 and refs. 1–23. [file 41563_2023_1792_MOESM1_ESM.pdf]

nature materials

Article https://doi.org/10.1038/s41563-023-01792-x

Chemo-mechanical failure mechanisms of the silicon anode in solid-state batteries

In the format provided by the authors and unedited

Supplementary information

This PDF file includes:

Computational details

Chemo-mechanical phase-field model

Supplementary Fig. 1–26

Supplementary Note 1–4

Supplementary Table 1–8

Supplementary References 1–23

Supplementary information: Computational details

The atomistic simulations were conducted using the density functional theory (DFT) framework as implemented in the Vienna *Ab Initio* Simulation Package (VASP). The projector augmented-wave pseudopotentials were used to describe the interaction between ions and electrons, and the exchange-correlation effects were treated using the Perdew–Burke–Ernzerhof (PBE) functional under the generalized gradient approximation (GGA).[1] Herein, the electronic configurations for the PAW potentials were $1s^2 2p^0$ for Li, $3s^2 3p^3$ for P, $3s^2 3p^4$ for S, $3s^2 3p^5$ for Cl and $3s^2 3p^2$ for Si. The VESTA package was used to visualize the various structures.[2]

Stable structure of LPSCl

Li atoms partially occupy the *48h* site in LPSCl, and LPSCl exhibits anti-site S/Cl disorder.[3] The *Supercell* code was utilized to tackle the issue of partial occupancy and S/Cl disorder. It has been demonstrated to be a powerful tool to enumerate possible ordering phenomena.[4-6] An exhaustive search over all possible configurations performed by the *Supercell* code for medium-size systems makes it possible to describe in-depth the distribution of local composition and resulting local geometrical distortions, with random as well as non-random types of disorder.[7] After enumerating the possible structures, Coulomb energy calculations were additionally performed to sort out 10 structures for LPSCl. The structure optimizations based on DFT calculations were then performed, and the structure with the lowest energy was selected for the subsequent calculations. The most stable LPSCl structure was screened in our previous study,[8] which was directly selected to calculate the adsorption energy.

Adsorption of Li atoms on Si and LPSCl surfaces

The most stable (100) and (111) surfaces were selected for LPSCl and Si in the subsequent calculations, respectively. The vacuum space in the *z* direction was about 15 Å to avoid interaction between neighboring slabs. The energy cut-off is set to 500 eV for the plane-wave expansion of the projector-augmented waves in the self-consistent calculations, whereas the Gamma grid of 1 × 1 × 1 was used. Each system was fully relaxed with residual forces smaller than 0.01 eV Å^{-1} and the total-energy change was less than 10^{-5} eV during the structural optimization. The binding energy of a lithium atom on a surface was determined using the expression,

$$E_{\mathrm{B}} = E_{\mathrm{Li}} + E_{\mathrm{surface}} - E_{\mathrm{total}} \quad (1)$$

where E_{B}, E_{Li}, E_{surface} and E_{total} represent the binding energy, the energy of a Li atom, of the Si (111) and LPSCl (100) surfaces, and of the adsorbate, respectively.

Melt-and-quench simulations

The “melt-and-quench” simulation scheme was conducted by carrying out a series of *ab-initio* molecular dynamics (AIMD) simulations.[9] The relaxed supercell equilibrated at 300 K for 2 ps, and was then heated to a temperature (2100 K) in 5 ps. The system was allowed to equilibrate at 2100 K for 2000 steps (each step = 1 fs). The process was then reversed to quench the system back to 300 K at rate of 300 K ps^{-1}. At each step, the structure obtained from the previous AIMD simulation was used as the starting point

for the next one. For each AIMD simulation, the system was given 2 ps to reach its thermal equilibrium in order to eliminate its correlation to the previous structure.

AIMD simulations for ionic conductivities of Li_xSi alloys

The diffusivity of Li^+ ions in amorphous Li-Si alloys was determined based on AIMD simulations with non-spin polarized calculations. Because AIMD simulations are computationally expensive, a compromise was made between computation efficiency and accuracy, resulting in the use of a kinetic energy cut-off of 300 eV and a *gamma*-point sampling.[10] The initial temperature of the amorphous Li-Si alloys was set to be 100 K, and the velocity of ions was set according to the Boltzmann distribution. Then, the samples were heated to the desired temperature (500 to 1200 K) using a velocity scaling thermostat at a heating rate of 1 K fs^{-1}. The total simulation time was set to 30 ps. The integration of Newton's equation was treated based on the Verlet algorithm, as implemented in VASP.[1] At the assigned temperature, the AIMD simulation was performed in the NVT ensemble[11] in conjunction with a Nose-Hoover thermostat.[11,12] The diffusion coefficient of the Li^+ ions was calculated by the mean square displacement (MSD) over time as

$$D_{\mathrm{Li}^+} = \frac{1}{2Nd\Delta t}\sum_{i=1}^{N} < [r_i(t+\Delta t) - r_i(t)]^2 > \quad (2)$$

where d is the dimensionality of diffusion, N is the total number of diffusing ions, $r_i(t)$ is the displacement of the i-th ion at time t. The activation energy (E_a) is determined from Arrhenius plots of the diffusion coefficient. The Li^+ ion conductivity was then calculated according to the Nernst-Einstein relationship,

$$\sigma_{\mathrm{Li}^+} = \frac{nq^2}{k_B T} D_{\mathrm{Li}^+} \quad (3)$$

where n is the charge carrier concentration of mobile ions as number density, q is the ionic charge (i.e., e_0), k_B is the Boltzmann constant and T is the temperature.[10]

BoltzTrap calculations for electronic conductivities of Li_xSi alloys

BoltzTraP is a modern implementation of the smooth Fourier interpolation algorithm for electronic bands, which forms the basis of the original and widely used BoltzTraP code.[13] The electron conductivity was calculated using BoltzTrap, which was carried out through Fourier expansion of energy bands and special function processing to maintain the symmetry of the space group. The amorphous structures of $LiSi_3$, LiSi, $Li_{12}Si_7$, $Li_{13}Si_4$, Li_7Si_2, $Li_{13}Si_4$, Li_7Si_2 and $Li_{15}Si_4$ were all selected for these calculations. Although the relaxation time τ can be defined by estimating the electron-phonon coupling effect, the calculations of the full electron-phonon interactions are complicated. The carrier (electron) relaxation time has been demonstrated to be ~ 10^{-14} s in the Materials Project.[14] The electron concentration has been also determined from the Bader charge

analysis, which has been widely used in calculating the charge transfer in various materials. The electron concentration (n) was calculated by:

$$n = C/V \tag{4}$$

Where n is the electronic concentration, C is the total transferred charge and V is the volume of the amorphous structure.

Supplementary information: Chemo-mechanical phase-field model

The schematic in **Supplementary Fig. 20** shows the model-type electrode for the chemo-mechanical phase-field model, consisting of the silicon anode and the LPSCl electrolyte, with the chemical reaction for the charge/discharge process taking place at the interface between the Si and the SE. The insertion of lithium into the Si causes a volume change, leading to the emergence of stress and elasto-plastic deformation. Hence, the free energy landscape for the chemo-mechanically coupled electrode scenario can be expressed as follows:[15-17]

$$\psi = \psi_{\mathrm{che}} + \psi_{\mathrm{ela}} + \psi_{\mathrm{d}} \tag{5}$$

where ψ_{che} represents the chemical free energy that accounts for the energy contribution due to the chemical reaction, i.e., the lithiation/delithiation reaction. ψ_{ela} is the elastic strain energy due to the elastic lattice deformation that originates from the injection and removal of the lithium, and ψ_{d} denotes the damage density energy for the crack formation and propagation. Given that Li^+ ions diffuse through the SE phase and are inserted (together with the necessary number of electrons) into the anode, then their contribution to the chemical free energy can be defined as follows:[18-20]

$$\psi_{\mathrm{che}} = RTC_{\mathrm{max}}[\mathrm{c}_{\mathrm{Li}}\ln(c_{\mathrm{Li}}) + \mathrm{c}_{\mathrm{Li}^+}\ln(c_{\mathrm{Li}^+})] \tag{6}$$

with R and T being the gas constant and temperature, and C_{max} is the maximum concentration of lithium stored in the Si anode, with the unit of mol/m^3. c_{Li} and c_{Li^+} are the normalized concentrations (normalized by $\mathrm{C}_{\mathrm{max}}$) of lithium in the Si anode and Li^+ ions in the SE, respectively, which are considered to assume an ideal solution state as reflected by Eq. 6 for the configurational entropy. Moreover, it is assumed that the electrons are continuously provided throughout the reaction, and thus their energy contribution is disregarded in this investigation. The potential change resulting from the change in electric neutrality due to the movement of anions and cations is not within the scope of this study and will be addressed in future research. The elastic free energy is given by:

$$\psi_{\mathrm{ela}} = \frac{1}{2}\boldsymbol{\sigma}:\boldsymbol{\varepsilon}_{\mathrm{e}} = \frac{1}{2}\left(\mathbb{C}(c_i,d):\left(\boldsymbol{\varepsilon} - \frac{(c_i - c_{\mathrm{ref}})\Omega}{3}\boldsymbol{I}\right)\right):\left(\boldsymbol{\varepsilon} - \frac{(c_i - c_{\mathrm{ref}})\Omega}{3}\boldsymbol{I}\right) \tag{7}$$

where $\boldsymbol{\sigma}$ is the Cauchy stress tensor and $\boldsymbol{I}$ is the identity tensor, $\boldsymbol{\varepsilon}$ and $\boldsymbol{\varepsilon}_{\mathrm{e}}$ are the total

strain and the elastic strain, respectively. c_i is the concentration of the i-th species, and c_{ref} is its reference concentration, Ω is the partial molar volume. $\mathbb{C}$ is the elasticity tensor which depends on both the concentration of the species as well as the damage state variable d. The damage density energy is read as follows:[21]

$$\psi_{\mathrm{d}} = \frac{d^2}{2G_{\mathrm{c}}l_0} + \frac{G_{\mathrm{c}}l_0}{2}|\nabla d|^2 \tag{8}$$

where G_c is the fracture energy, and l_0 is the length scale parameter of the crack. In this work, $d = 1$ represents the fully damaged case and $d = 0$ denotes the unbroken fully coherent case, respectively.

The equations that govern the diffusion of each species, the linear momentum balance, and the propagation of cracks are expressed as follows:

$$\frac{\partial c_i}{\partial t} = \nabla \cdot (D_i RTc\nabla \mu_i) + \nu_i \dot{r}(c_i), with\ i \in \{\mathrm{Li}, \mathrm{Li}^+\} \tag{9}$$

where c_i is the normalized concentration (divided by C_{max})of i-th species, and its chemical potential is given by $\mu_i = \frac{\partial \psi}{\partial c_i}$, which contains the contribution from both the diffusion and mechanical deformation. D_i is the diffusion coefficient of i-th species. R is the gas constant, and T is the temperature. $\dot{r}$ is the reaction source term, and ν_i is the stoichiometric coefficient of i-th species.

$$\nabla \cdot \sigma = 0 \tag{10}$$

holds for the linear momentum balance.

An isotropic J2 plasticity model is employed for the inelastic plastic response of system, with the yield condition and plastic flow expressed as follows:[22]

$$f = \|dev[\boldsymbol{\sigma}]\| - \sqrt{\frac{2}{3}}\left(\sigma_Y + He_p\right) \tag{11}$$

$$\dot{\boldsymbol{\varepsilon}}_p = \lambda \frac{\partial f}{\partial \boldsymbol{\sigma}} = \lambda \frac{\boldsymbol{\sigma}}{\|dev[\boldsymbol{\sigma}]\|} \tag{12}$$

$$\dot{e}_p = \lambda \sqrt{\frac{2}{3}} \tag{13}$$

where σ_Y and e_p represent the yield stress and equivalent plastic strain, respectively. λ is the unknown plastic multiplier to be solved during the simulation. $dev[\boldsymbol{\sigma}]$ is the deviatoric part of the Cauchy stress.

The crack propagation is governed by

$$\frac{\partial d}{\partial t} = -L\frac{\delta\psi}{\delta d} \quad (14)$$

where L is the mobility for the crack propagation, and the variational derivative is defined as: $\frac{\delta\psi}{\delta d} = \frac{\partial\psi}{\partial d} - \nabla\cdot\frac{\partial\psi}{\partial d}$. (15)

In this work, the kinetics of the lithium insertion and extraction is given by the Butler-Volmer equation as follows:

$$\dot{r} = k_0[\exp\left(\frac{F\eta}{2RT}\right) - \exp\left(-\frac{F\eta}{2RT}\right)] \quad (16)$$

with k_0 being the reaction coefficient, and η being the overpotential. The parameters for the phase-field model are listed in **Supplementary Table 8**.

It is important to note that the presented chemo-mechanical phase-field model is a general model, and can be applied to both 2D and 3D modeling. However, in this study, we have focused on the use of 2D geometry to examine the differences between different types of interfaces, such as the 2D and 3D interfaces.

Supplementary Information: Additional figures and tables

Supplementary Note 1

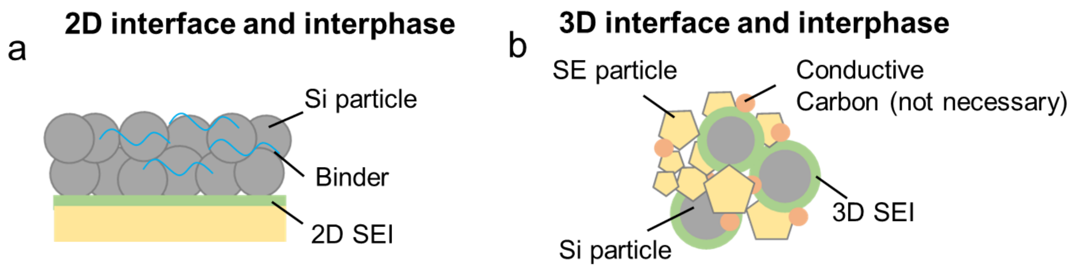

Supplementary Fig. 1 Comparison between 2D and 3D interfaces. Schematic of (a) 2D interface and interphase of a compact Si anode and (b) 3D interface and interphase of a Si/SE/C composite.

We like to note that real-world interfaces are rarely true 2D interfaces, and that one might consider them rather as quasi-2D interfaces – in particular when it comes to SEI formation. Distinguishing "2D" from "3D", we like to highlight that a SE-free Si anode shows only one more or less planar SEI layer between Si and SE, while the Si composite anode shows an interconnected 3D network of the SEI. As **Supplementary Fig.1a** shows, the SEI grows on the SE-free Si anode as a thin layer into the LPSCl separator, forming a "2D SEI" during cycling. Thus, with "2D" we like to note the planar interphase character of the SEI. In contrast, the "3D SEI" describes the SEI grown in an interconnected 3D network as part of the Si/LPSCl/C composite (**Supplementary Fig.1b**).

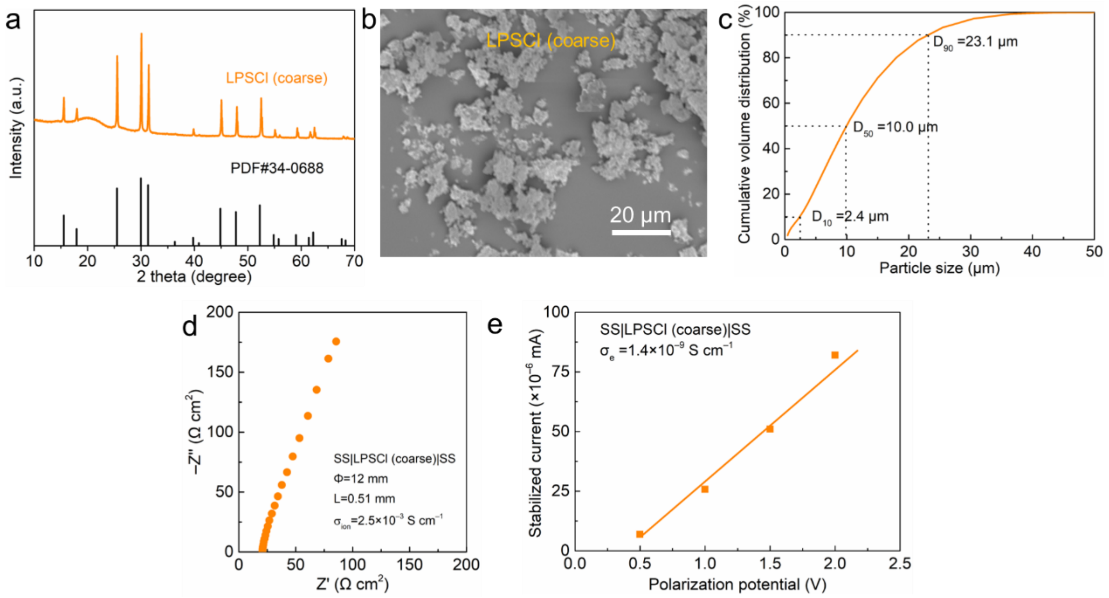

Supplementary Fig. 2. Properties of the LPSCl (coarse). (a) XRD pattern, (b) SEM image, and (c) particle size distribution of LPSCl (coarse) particles. (d) Impedance of a LPSCl (coarse) pellet measured by EIS. (e) Electronic conductivity of a LPSCl (coarse) pellet measured by DC polarization. 380 MPa was applied to compress the powder followed by a constant pressure of 50 MPa during the impedance measurement and DC polarization at 25 ºC.

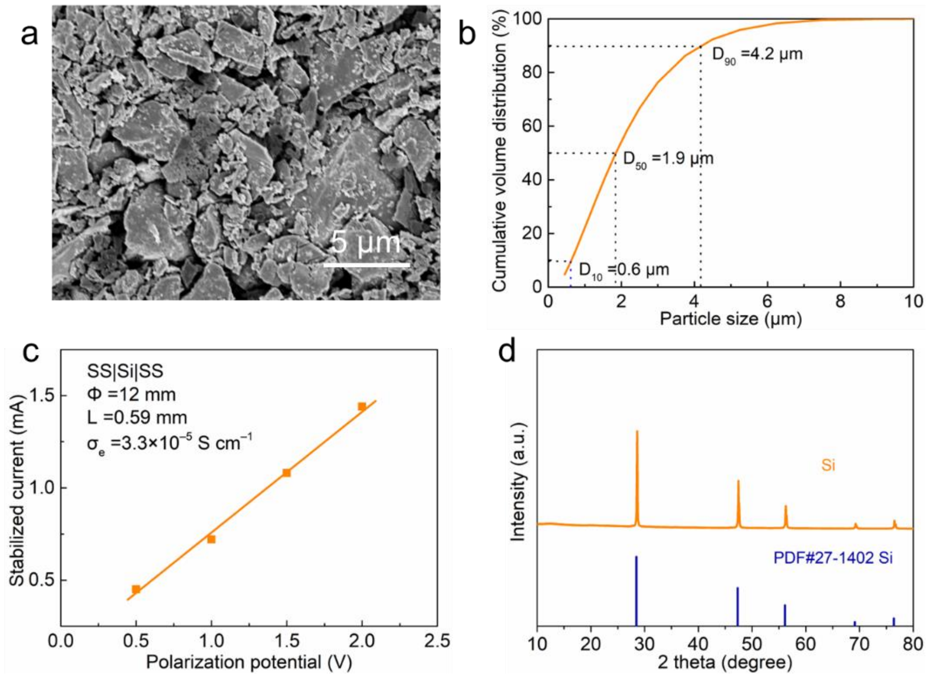

Supplementary Fig. 3. Properties of Si particles. (a) SEM image and (b) particle size distribution of Si particles. (c) Electronic conductivity of a Si pellet measured by DC polarization. (d) XRD pattern of Si particles. 380 MPa was applied to compress the powder followed by a constant pressure of 50 MPa during the DC polarization at 25 ºC.

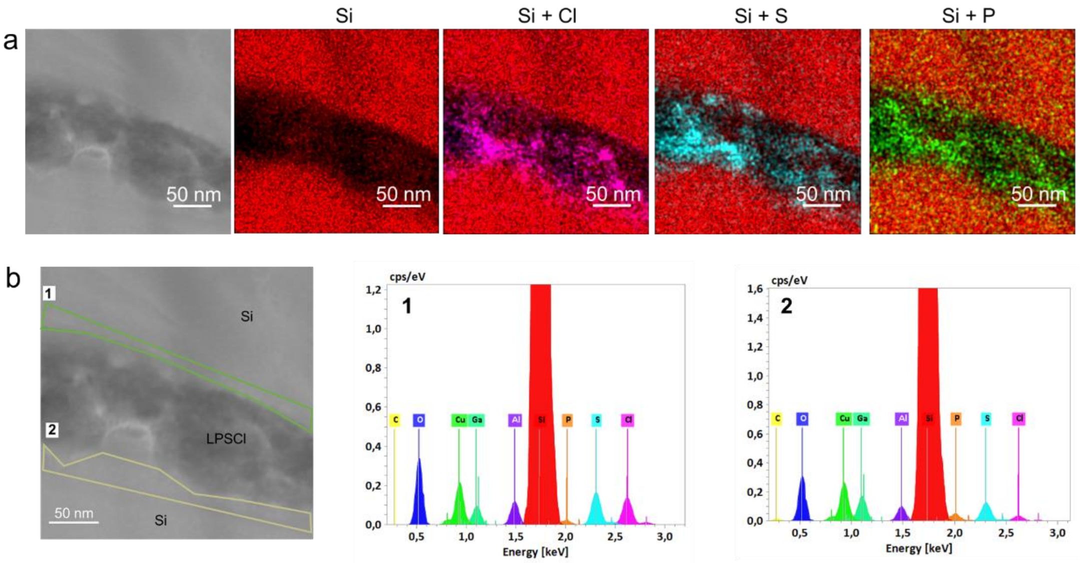

Supplementary Fig. 4. TEM data. (a) HAADF STEM image of the Si/LPSCl sample and corresponding EDS maps. (b) EDS profiles at the corresponding interface area. Cu and Ga signals originate from the Cu mesh and Ga ion beam in FIB, respectively. Al signal may be from the impurity.

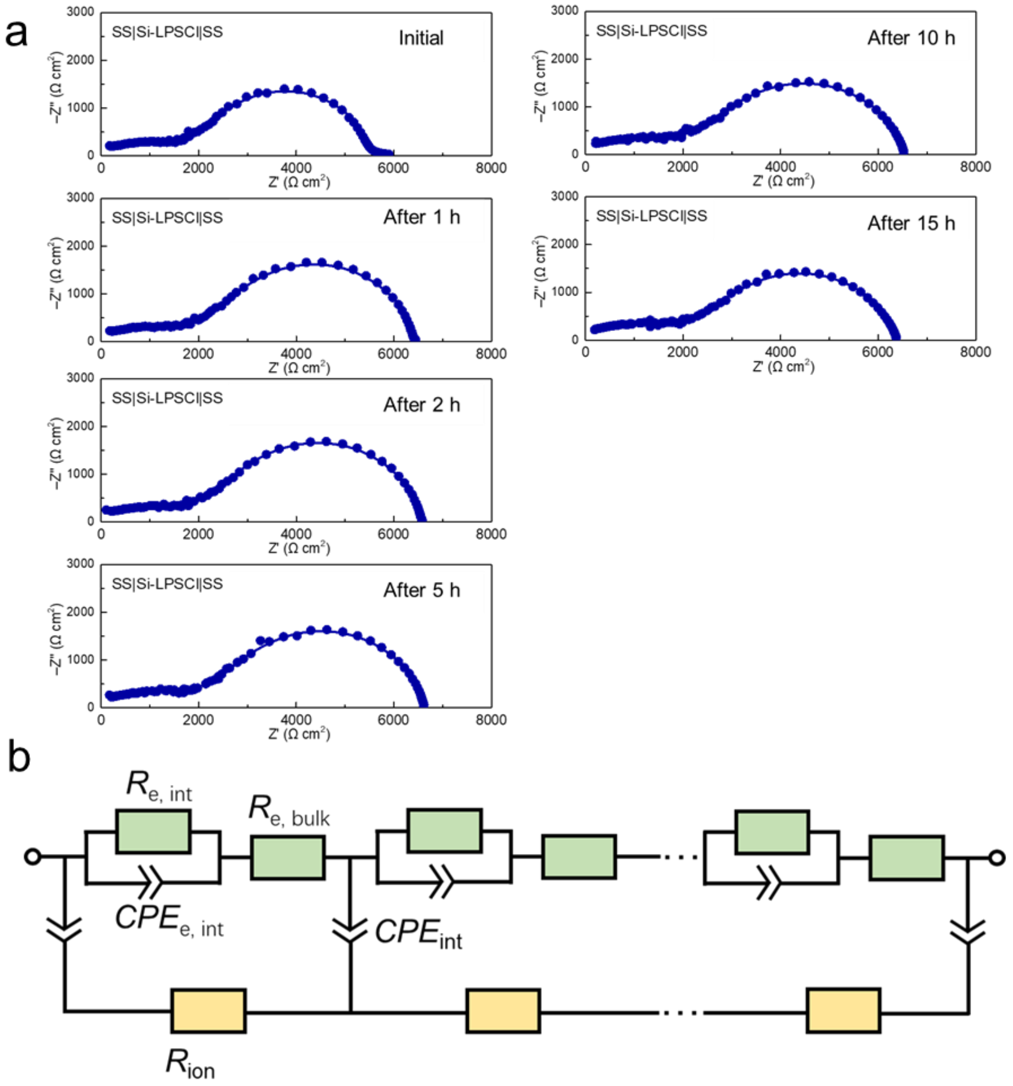

Supplementary Fig. 5. Impedance of pristine Si/LPSCl composites. (a) Impedance evolution of the Si/LPSCl composite with time. (b) Transmission line model used for fitting the impedance spectra. The two resistor elements $R_{e,bulk}$ and $R_{e,int}$ in the electronic branch (green) are attributed to electronic bulk transport and interfacial charge transfer, respectively. The resistor element R_{ion} in the ionic branch (yellow) is attributed to ionic bulk transport. The fit data is shown in **Supplementary Table 1**.

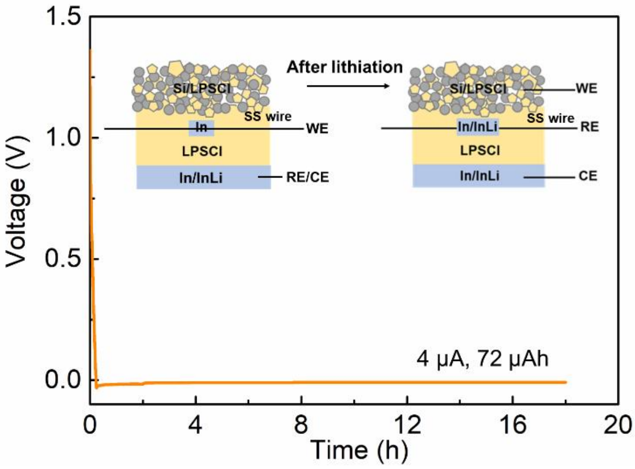

Supplementary Fig. 6. Lithiation of the In wire as reference electrode for the three-electrode cell. A 0.8 mg (thickness =10 μm) In foil rolled on a thin stainless-steel wire (thickness = 80 μm) was buried in the middle of LPSCl pellet. The In/InLi anode was used to provide Li for the lithiation of the In "wire". The small In/InLi "wire" serves after lithiation as the reference electrode, while the working electrode (i.e. Si/LPSCl) and counter electrode (i.e. In/InLi foil) in three-electrode cells are the same as those in two-electrode cells.

Supplementary Note 2

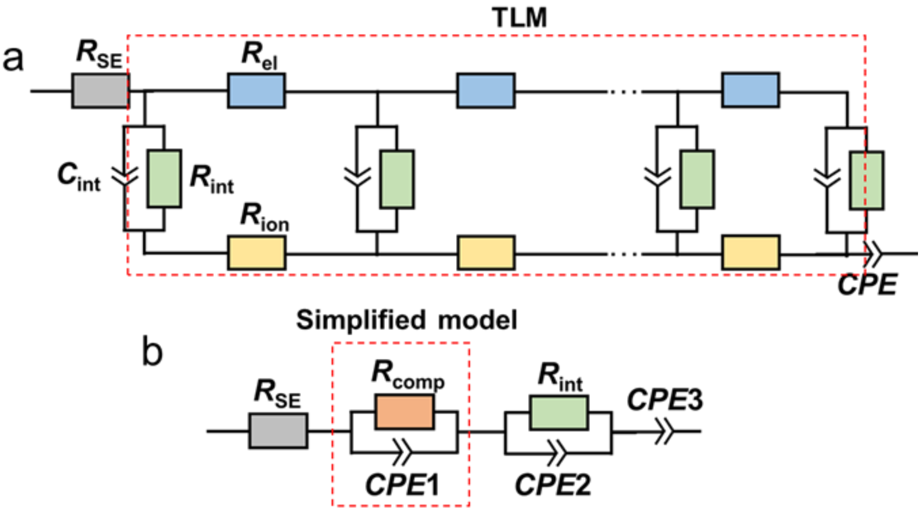

Supplementary Fig. 7. Different models for the EIS fitting. (a) TLM and (b) simplified model for the EIS fitting.

The transmission line model (TLM) in **Supplementary Fig. 7a** is thought for the EIS fitting of the mixed-conducting Si/LPSCl composites, which show parallel ionic and electronic paths. For example, we applied a similar TLM to investigate the ionic and electronic conductivity of composite Si/LPSCl anodes before lithiation (**Supplementary Fig. 5**), where the electron transport is along the Si phase and ion transport is along the LPSCl phase. Different from the separated ionic and electronic paths in the Si/LPSCl anodes before lithiation, the Li_xSi in the Si/LPSCl anodes after lithiation

shows mixed conduction. It does not only conduct electrons, but also conducts ions together with the LPSCl phase. The resulting complex electronic/ionic paths are not well described by the TLM. Therefore, a simplified model in **Supplementary Fig. 7b** was used, where R_{ion} and R_{el} are combined into R_{comp}. Since we focus on the R_{int} change to study the SEI growth rate, the simplified model works well for both 3D and 2D Si/LPSCl interfaces.

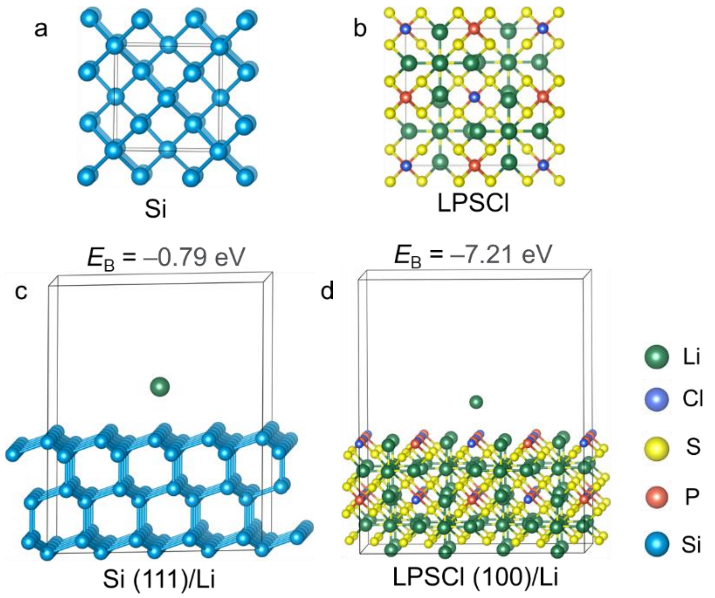

Supplementary Fig. 8. Comparison of the binding energy. Schematic structures of (a) bulk Si and (b) bulk LPSCl. (c) Sketch of the model for the calculation of the binding energy between Si (111) and Li. (d) Sketch of the model for the calculation of the binding energy between LPSCl (100) and Li.

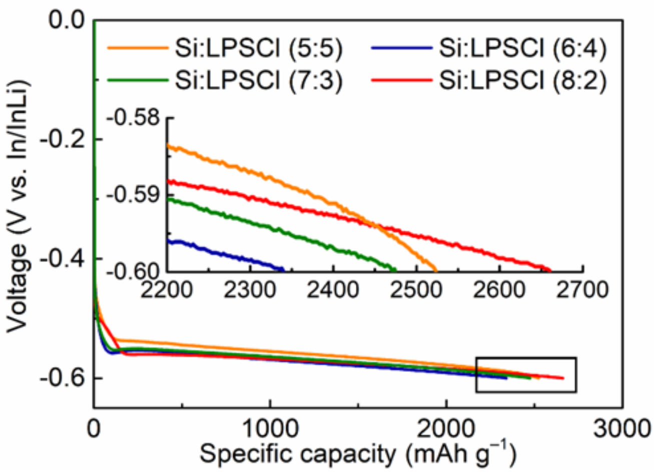

Supplementary Fig. 9. Specific capacities of different Si/LPSCl anodes. Lithiation curves of different Si/LPSCl anodes at 0.1 C.

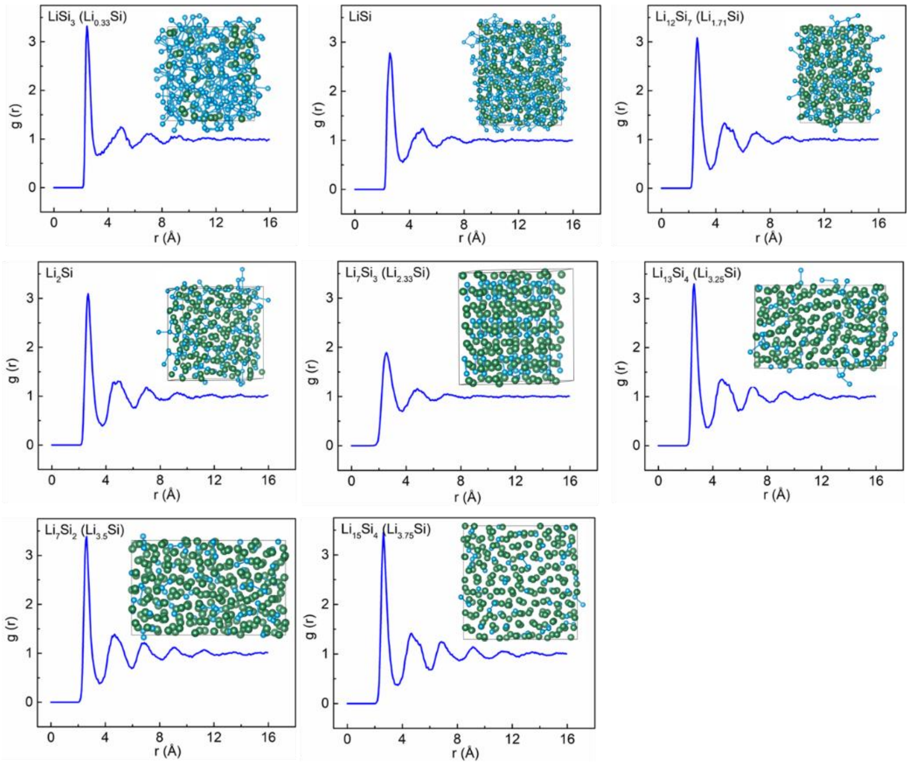

Supplementary Fig. 10. Structure of Li$_x$Si alloys. The radial distribution function for amorphous Li$_x$Si alloys with corresponding amorphous structures.

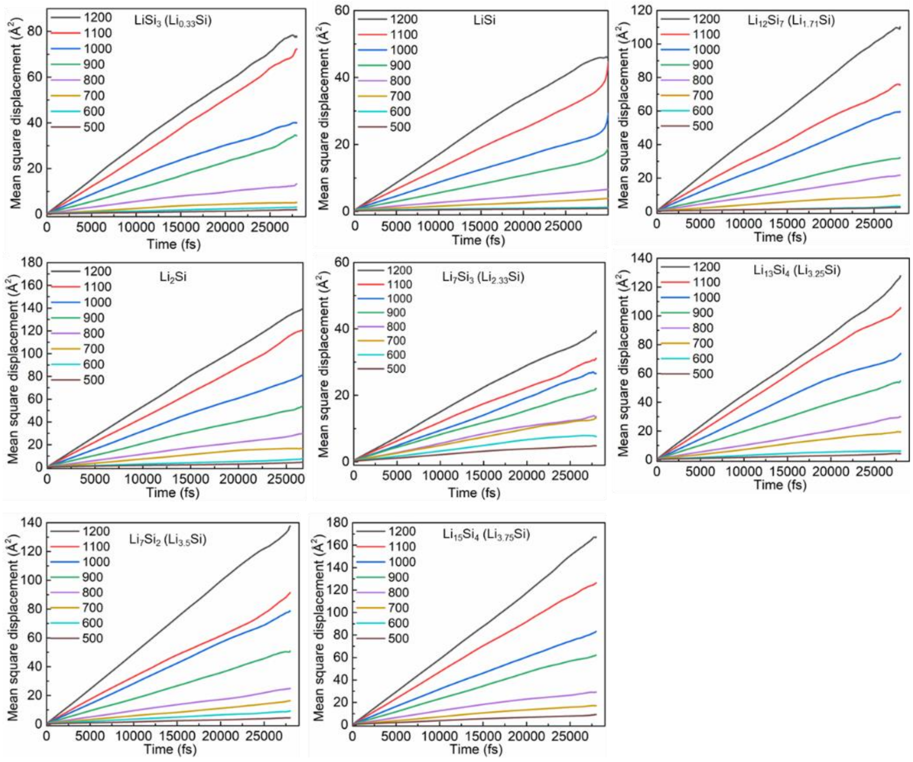

Supplementary Fig. 11. AIMD simulation of lithium diffusion. Mean square displacement of lithium atoms in amorphous Li$_x$Si structures at different temperatures (in K) for the calculation of activation energies.

Supplementary Note 3

The partial ionic/electronic conductivities obtained by DFT simulations are consistent with the $\widetilde{D}_{\mathrm{Li}}$ values and the thermodynamic factor obtained by GITT measurements, further confirming the accuracy of the $\widetilde{D}_{\mathrm{Li}}$ values from the GITT measurements. $\widetilde{D}_{Li}$ can be calculated by the following equations:

$$\widetilde{D}_{\mathrm{Li}} = D_{\mathrm{Li}} \cdot W \qquad (17)$$

$$D_{\mathrm{Li}} = \frac{D_{\mathrm{Li}^+} \cdot D_{\mathrm{e}^-}}{D_{\mathrm{Li}^+} + D_{\mathrm{e}^-}} = \frac{\sigma_{\mathrm{Li}^+} \cdot \sigma_{e^-}}{\sigma_{\mathrm{Li}^+} \cdot c_{\mathrm{e}^-} + \sigma_{\mathrm{e}^-} \cdot c_{\mathrm{Li}^+}} \cdot \frac{RT}{F^2} \qquad (18)$$

D_{Li} is the lithium (component) diffusion coefficient, i.e., the self-diffusion coefficient of the neutral component Li. W is the thermodynamic factor. σ_{Li^+} and σ_{e^-} are the partial ionic and electronic conductivities of the considered phase. R, T, and F are the gas constant, temperature, Faraday's constant, respectively. We can safely assume for the Li-Si alloys that

$$c_{\mathrm{Li}^+} \cong c_{\mathrm{e}^-} \cong c_{\mathrm{Li}} \qquad (19)$$

where c_{Li^+}, c_{e^-}, and c_{Li} are the concentrations of Li^+, e^-, and Li (i.e., the neutral component Li^0) in the $\mathrm{Li}_x\mathrm{Si}$ alloy.

The average value of $\widetilde{D}_{\mathrm{Li}}$ for a given concentration range results as

$$\overline{\widetilde{D}}_{\mathrm{Li}} = \overline{D}_{\mathrm{Li}} \cdot \overline{W} = \frac{1}{\bar{c}_{\mathrm{Li}}} \cdot \frac{\bar{\sigma}_{\mathrm{Li}^+} \cdot \bar{\sigma}_{\mathrm{e}^-}}{\bar{\sigma}_{\mathrm{Li}^+} + \bar{\sigma}_{\mathrm{e}^-}} \cdot \frac{RT}{F^2} \cdot \overline{W} \qquad (20)$$

where $\overline{W} = 3.0$, $\bar{\sigma}_{\mathrm{Li}^+} = 1.5\times10^{-3}$ S cm^{-1}, and $\bar{\sigma}_{\mathrm{e}^-} = 4.4\times10^{-4}$ S cm^{-1}, respectively (data taken from results in Fig. 3d, 3e, and Supplementary Fig.19c). We also assume

$$\frac{1}{\bar{c}_{\mathrm{Li}}} \cong \frac{V_{\mathrm{m}}}{X_{\mathrm{Li}}} \text{ based on } \mathrm{Li_2Si}\text{, i.e. } x_{\mathrm{Li}} = 2, \qquad (21)$$

where V_{m} and x_{Li} are molar volume and molar number of $\mathrm{Li_2Si}$, respectively. Then,

$$\overline{\widetilde{D}}_{\mathrm{Li}} = 31.3 \cdot \frac{1.5\times10^{-3} \cdot 4.4\times10^{-4}}{1.5\times10^{-3} + 4.4\times10^{-4}} \cdot \frac{8.314\times300}{96485.3^2} \cdot 3.0 \text{ cm}^2\text{s}^{-1} = 8.6\times10^{-9} \text{ cm}^2\text{s}^{-1},$$

which is in excellent agreement with the $\overline{\widetilde{D}}_{\mathrm{Li}} = 1.0\times10^{-8}$ cm^{2}s^{-1} as obtained from the GITT measurements. We note that this analysis of chemical diffusion of Si, including the comparison of theoretical and experimental data has not been demonstrated before to the best of our knowledge. However, it is well in line with the classical analysis of chemical diffusion in other lithium alloys (e.g. $\mathrm{Li_3Sb}$, and $\mathrm{Li_3Bi}$), as originally presented by Weppner et al.[23]

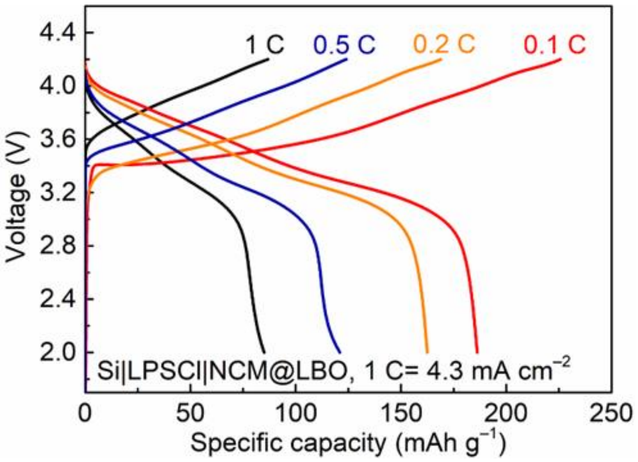

Supplementary Fig. 12. Sufficient ionic/electronic conductivity of lithiated SE-free Si anodes. Rate performance of the Si|LPSCl|NCM@LBO cells. The N/P ratio is 1.3.

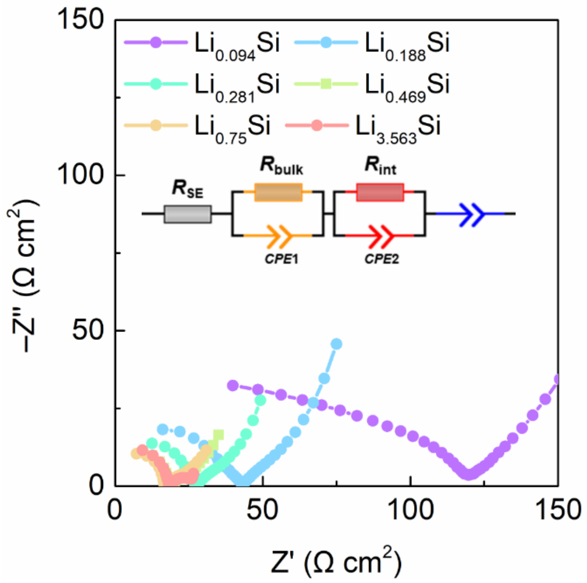

Supplementary Fig. 13. Impedance measurements during the relaxation of GITT. The inset shows the corresponding equivalent circuit used to evaluate the impedance data. The fit data are shown in **Supplementary Table 5**.

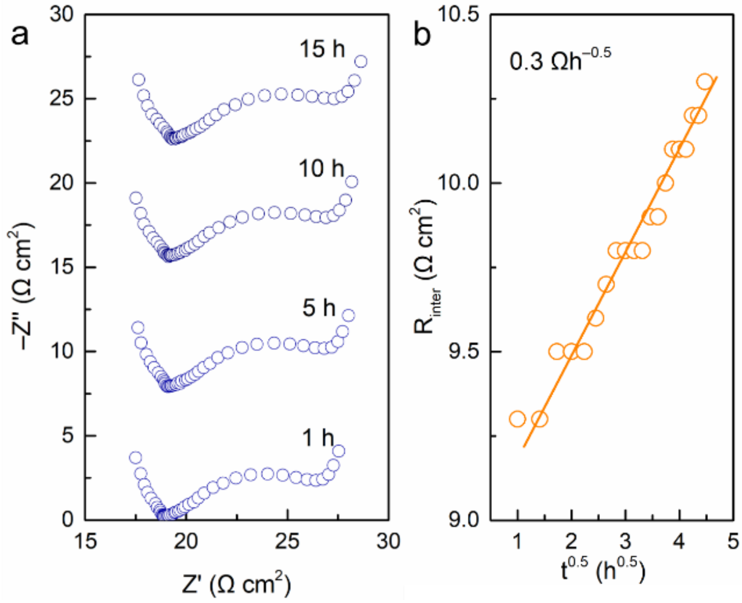

Supplementary Fig. 14. Kinetics of SEI growth at *E* = 0.02 vs. Li^+/Li. (a) Nyquist plots (WE vs. RE) of a three-electrode cell with long-term resting (WE: lithiated SE-free Si anode, RE: In/InLi, and CE: In/InLi). The three-electrode setup is shown in Supplementary Fig.5. (b) R_{int} at the 2D Si|LPSCl interface as a function of the square root of time ($t^{0.5}$). The fit data are shown in **Supplementary Table 6**.

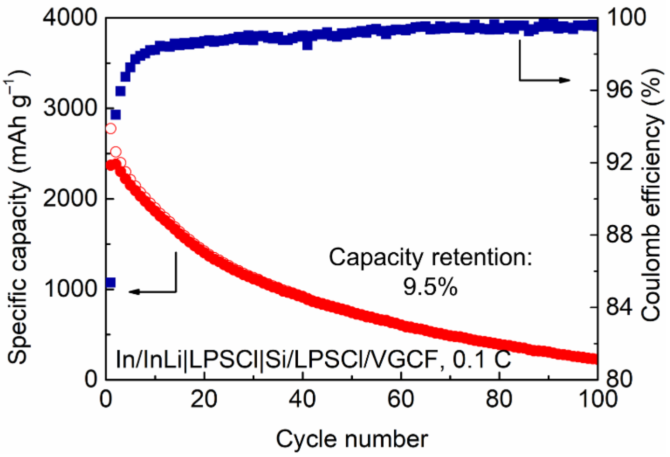

Supplementary Fig. 15. The effect of electronically conducting additives. Cycling performance of the In/InLi|LPSCl|Si/LPSCl/VGCF cells at 0.1 C and 50 MPa.

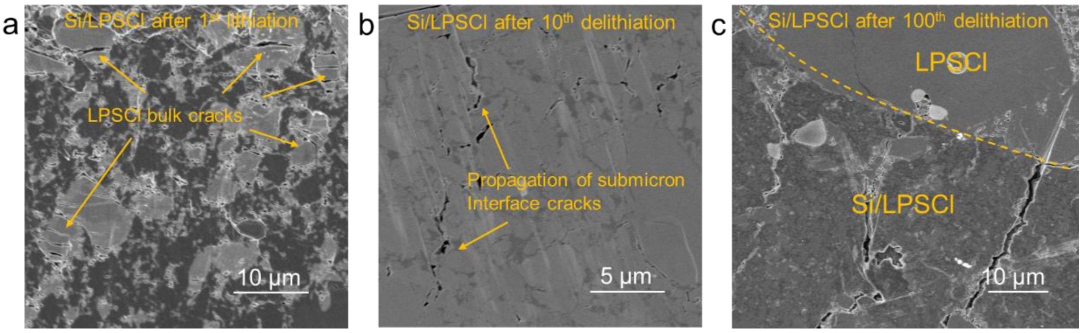

Supplementary Fig. 16. SEM analysis of Si/LPSCl composite anodes. Cross-sectional SEM images of the Si/LPSCl anode (a) after 1st lithiation at a low magnification, (b) after 10th delithiation and (c) after 100th delithiation at a low magnification.

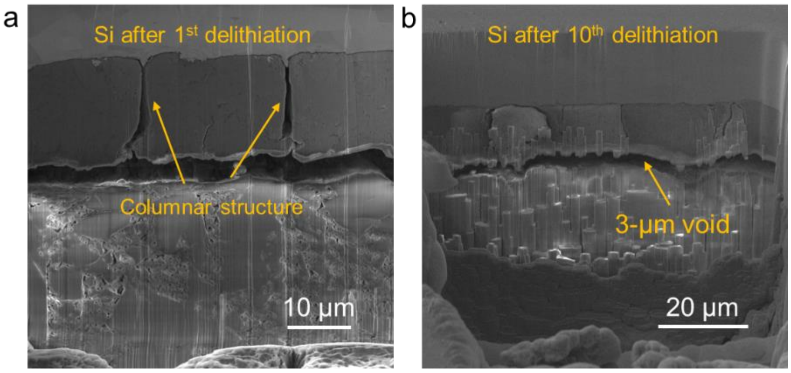

Supplementary Fig. 17. SEM analysis of SE-free Si anodes. Cross-sectional SEM images of the SE-free Si anode (a) after 1st delithiation at a low magnification, (b) after 10th delithiation.

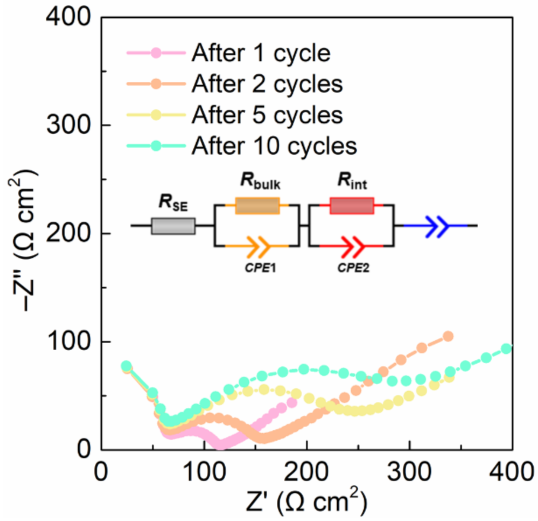

Supplementary Fig. 18. Impedance data of three-electrode cells with SE-free Si anode. Nyquist plots (WE vs. RE) of a three-electrode cell after different cycles. The inset shows the setup of the three-electrode cell. The fit data is shown in **Supplementary Table 7.**

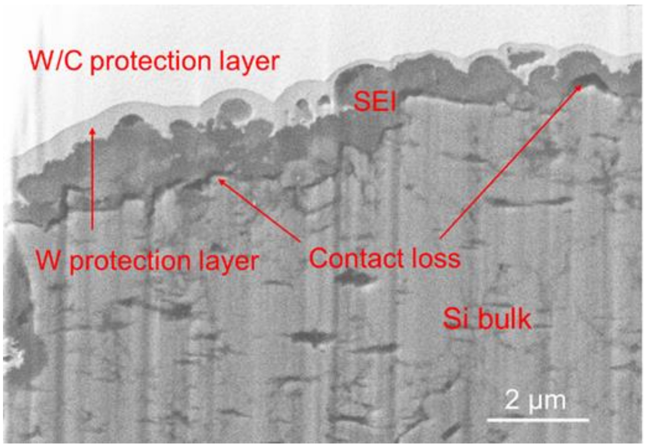

Supplementary Fig. 19. Stability of the SEI layer. Cryo-FIB-SEM image of SE-free Si anode after 1 cycle.

Supplementary Note 4

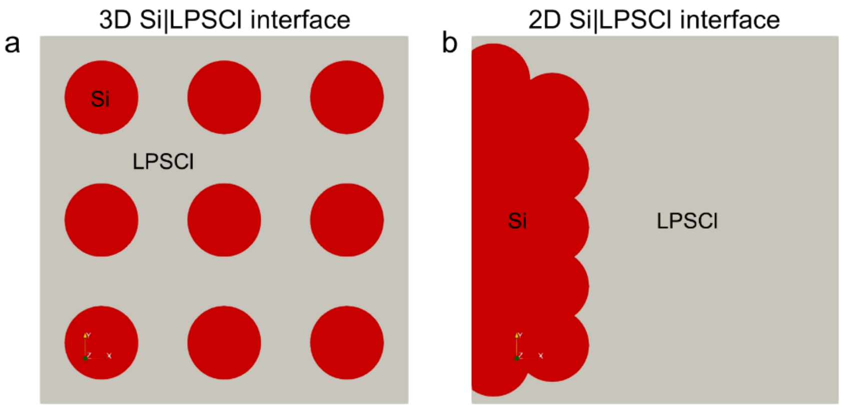

Supplementary Fig. 20. Geometry of chemo-mechanical phase-field models. (a) 3D Si/LPSCl composite and (b) 2D Si|LPSCl interface.

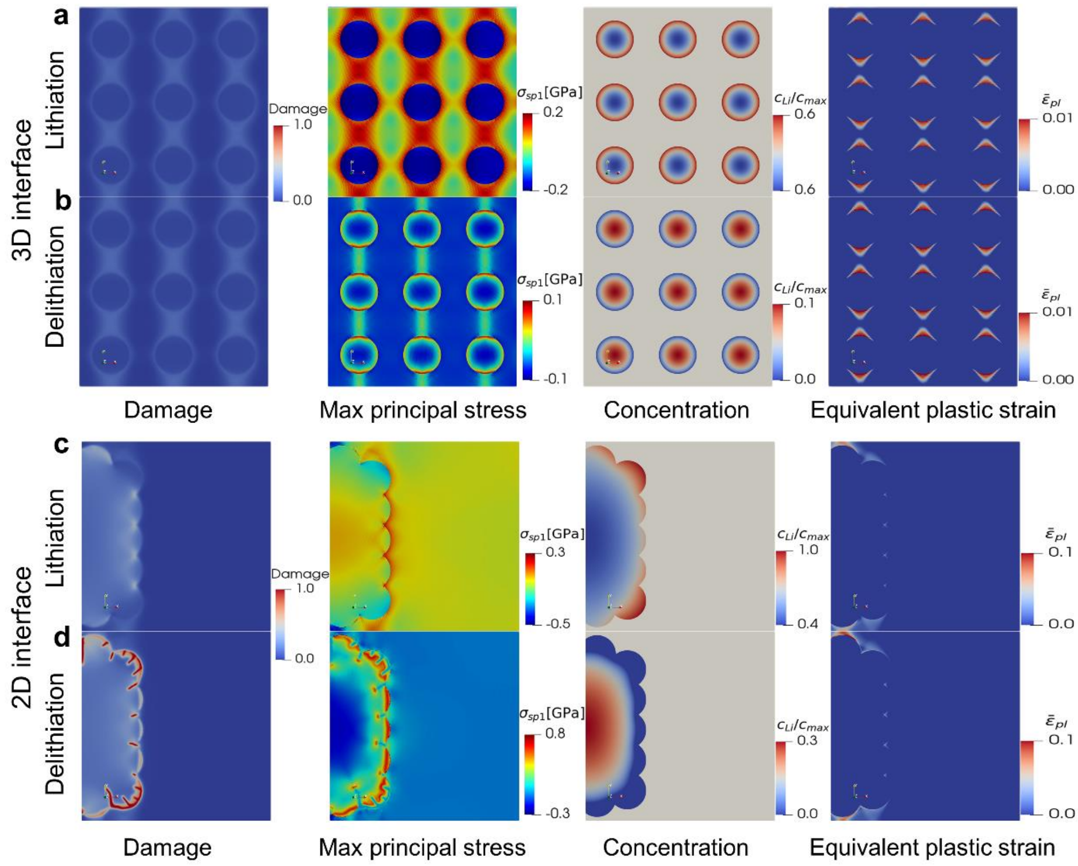

Supplementary Fig. 21 Phase-field fracture modeling of composite Si/LPSCl and SE-free Si anodes. Results from a phase-field model of the two different Si anodes. The distribution of cracks (damage), maximum principal stress, lithium concentration, and equivalent plastic strain after (a) 1st lithiation of the 3D Si/LPSCl composite, (b) 1st delithiation of the 3D Si/LPSCl composite, (c) 1st lithiation at the 2D Si|LPSCl interface, and (d) 1st delithiation at the 2D Si|LPSCl interface. The stack pressure is 50 MPa.

The charge and discharge steps obviously combine both chemical processes (lithium

chemical diffusion and storage) and mechanical processes (elasto-plastic deformation). To quantitatively understand the different elasto-plastic expansion/shrinkage of Si and the fracture behavior of LPSCl in 3D composites and at 2D LPSCl|Si interfaces, a fully coupled chemo-mechanical phase-field fracture model was developed to simulate the 1st lithiation and delithiation steps (**Supplementary Fig. 20**). The model is designed to be versatile and capable of modeling both 2D (circular particles) and 3D (spherical particles) systems. The 2D circular particle model has been used to demonstrate the differences between interfaces in the 3D composite and the case of the planar interface in the SE-free anode. In this model, both the Si and SE particles undergo elasto-plastic deformation, i.e., they exhibit distinct yield stresses and inelastic mechanical response together with linear elastic properties (i.e., Young's modulus). For further details, please refer to our supplementary material. The 3D interface, as shown in **Supplementary Fig. 21a and Fig. 21b**, exhibits a smooth connection between Si and SE. The smooth interface contact (the smooth circular surface) between the particle and SE is the reason behind the uniform distribution of stress (ranging from –0.2 to 0.2 GPa during lithiation and –0.1 to 0.1 GPa during delithiation) within and around the particle, thereby preventing the formation of noticeable cracks. This confirms that the lithiation process and the associated volume expansion of Si results in the disappearance of interface voids due to a "compression" effect and densification of the Li_xSi microstructure. Consequently, the lithium distribution within the Si particle is almost uniform throughout the (de)lithiation process (the lithium uniformly increases or decreases within the Si particle), with no significant change in the lithium concentration gradient from its core to the surface being captured, as the SoC increases from 5% to 60% and subsequently decreases from 60% to 10%, as shown in **Supplementary Fig. 21a and Fig. 21b**. Furthermore, during lithiation, the positive peak value (tensile stress around 0.2 GPa) of the maximum principal stress (σ_{sp_1}) is primarily observed at the surface of the particle due to the lithium insertion induced volume expansion. In contrast, during delithiation, the top and bottom of the particle are subjected to tensile stress around 0.1 GPa caused by the external pressure (compression from SE). In addition, a section of the SE matrix (located at the top and bottom parts attached to the particle) undergoes plastic deformation, resulting in an equivalent plastic strain of up to 1%. Throughout the entire cycle of lithiation and delithiation, cracks are not prominently visible (the damage percentage $d \ll 100\%$) as demonstrated in **Supplementary Fig. 21a and Fig. 21b**, owing to the low levels of stress. This indicates a good interconnection within the Li_xSi microstructure, as observed in **Fig. 4**.

The geometry of the 2D interface depicted in Fig. 5 creates stress concentration (due to the geometry singularity) features with stress peaks of up to 300 MPa (0.3 GPa) already upon the first lithiation at the anode/SE interface. The process of lithiation involves the insertion of lithium into the Si particle (the SoC reaches 100% at the end of the lithiation process), causes large volume expansion and exacerbates the stress at the singular interface, as shown in **Supplementary Fig. 21c**. As lithiation progresses from 5% to 100% (see **Supplementary Fig. 21a**), the principal stress σ_{sp_1}, which builds up due to

the growing misfit at the interface between the adjacent solid phases (i.e. LPSCl SE), reaches a range of approximately (–0.3~0.5 GPa). This value is higher than that observed in the 3D composite and can initiate the development of cracks and plastic flow of the lithiated Si. We expect that a stronger densification effect will occur at the 2D interface. Subsequently, upon delithiation, the removal of lithium (from 100% to 0%) causes the particles to shrink, as shown in **Supplementary Fig. 21d**. This, in turn, augments the stress at the 2D interface, ultimately leading to the propagation of cracks from the LPSCl into the Si particle, as depicted in **Supplementary Fig. 21d**. This further confirms the submicron cracks as observed in **Fig. 4**. The occurrence of fractures leads to a decrease in stress (stress relaxation) in the influenced zone (with a zero stiffness) as the cracks progress and stress levels are lowered to a zero-stress state. This results in a significant difference in stress levels between the damaged region and the undamaged domain. Towards the end of the delithiation process, the plastic deformation is further accumulated, therefore, σ_{sp_1} can climb up to –0.3~0.8 GPa, which is higher than that observed during the lithiation process. As a result, several large cracks are observed at the end of the delithiation process in the 2D interface, which is the primary cause of the large voids (around 2μm) depicted in **Fig. 4**. Furthermore, the surface of the Si particle is subjected to a negative principal stress (compressive stress indicated by the blue region), whereas the delithiation process can cause a positive principal stress (tensile stress) at the surface of the particle. Hence, as a result of the lithiation process, a considerable quantity of plastic strain is accumulated at the interface, which can be further increased by the delithiation process, as depicted in the bottom column of **Supplementary Fig. 21c and Fig. 21d**, by approximately 10%. This could be a significant contributing factor to the relatively stable porosity of the SE-free Si anode (~26.9%) after the first delithiation, as depicted in **Fig. 4**. Moreover, the value of the plastic strain is significantly greater than the value observed in the 3D composite (1%). Based on the findings in this section, it can be inferred that the 2D Si/LPSCl interface geometry shows stress accumulation, leading to the growth of cracks (voids) and undermines the cycling stability.

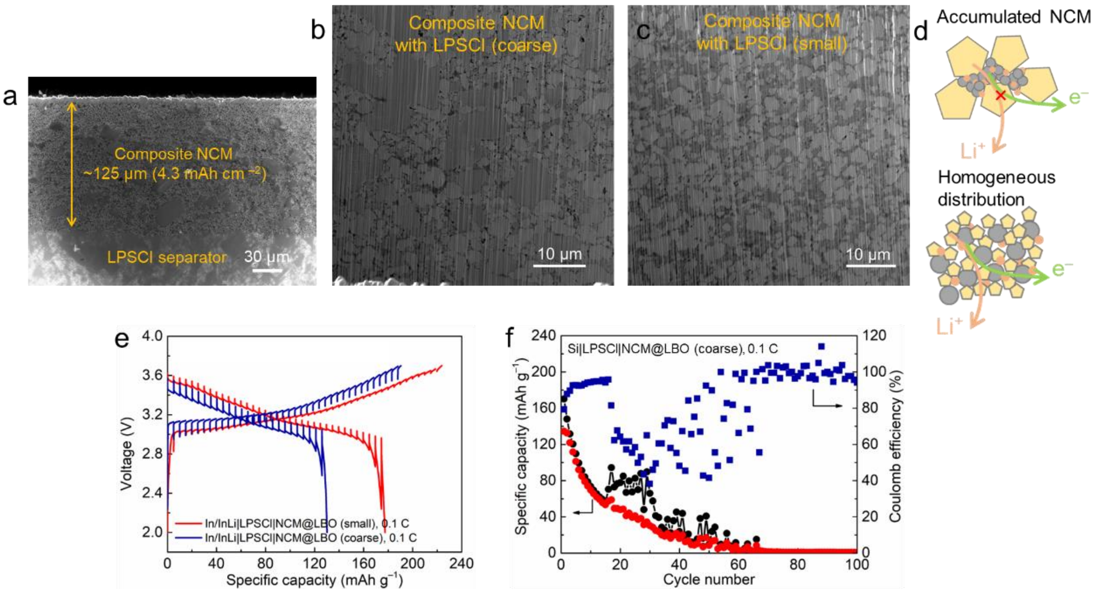

Supplementary Fig. 22. Results for the composite NCM cathode. (a) Cross-sectional SEM images of composite NCM cathode. Microstructures of composite NCM cathodes with (b) LPSCl (coarse) particles and (c) LPSCl (small) particles. (d) Schematic of ion/electron transport in the composite NCM cathodes with different LPSCl particles. (e) GITT curves of the In/InLi|LPSCl|NCM@LBO (small) cell and the In/InLi|LPSCl|NCM@LBO (coarse) cell at 0.1 C. (g) Cycling performance of the Si|LPSCl|NCM@LBO (coarse) cell at 0.1 C.

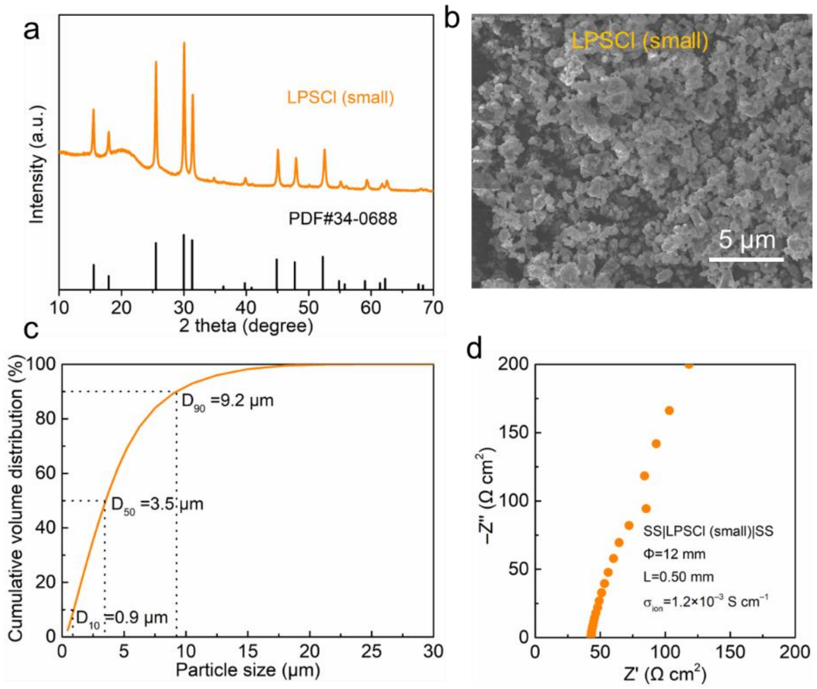

Supplementary Fig. 23. Properties of the LPSCl (small). (a) XRD pattern, (b) SEM image, and (c) particle size distribution of LPSCl (small) particles. (d) Impedance of a LPSCl (small) pellet measured by EIS. 380 MPa was applied to compress the powder followed by a constant 50 MPa during the impedance measurement at 25 ºC.

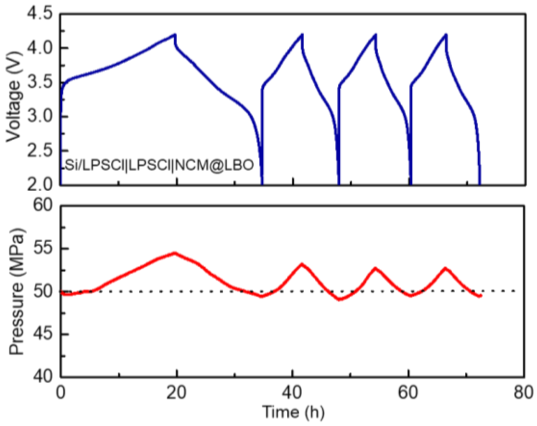

Supplementary Fig. 24 Galvanostatic cycling of the Si/LPSCl|LPSCl|NCM@LBO full cell along with the measured stack pressure changes. Four cycles were performed at 0.05 C for the first cycle and 0.1 C for the following cycles. The pressure $t = 0$ is 50 MPa.

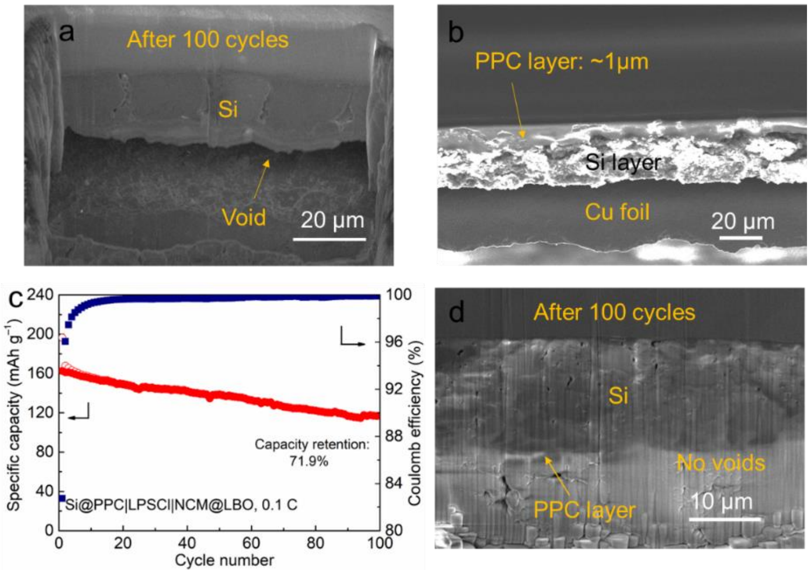

Supplementary Fig. 25. Additional experimental information on SE-free Si anodes. (a) Cross-sectional SEM image of the 2D Si|LPSCl interface in the Si|LPSCl|NCM@LBO cell after 100 cycles. (b) Cross-sectional SEM image of a Si@PPC sheet. (c) Cycling performance of the Si@PPC|LPSCl|NCM@LBO cell. (d) Cross-sectional SEM image of the 2D Si@PPC/LPSCl interface after 100 cycles.

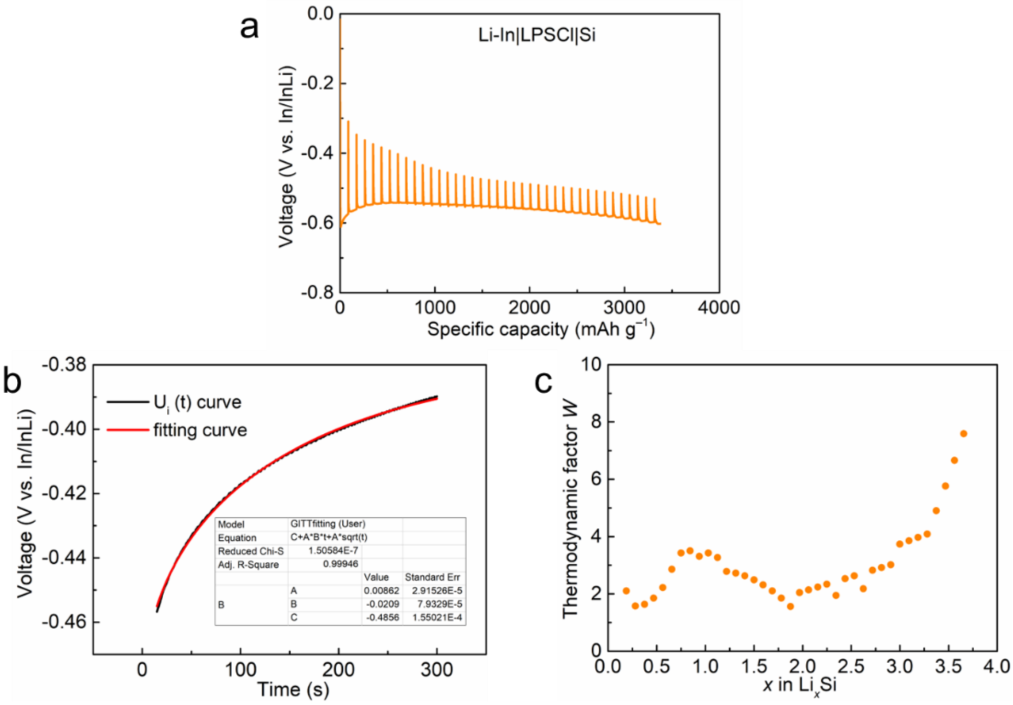

Supplementary Fig. 26. Coulometric titration of Si anode and thermodynamic factor. (a) GITT curve of the In/InLi|LPSCl|Si cell. (b) Fitting of relaxation potential U_i curve after first polarization (i.e. $Li_{0.094}Si$). (c) Thermodynamic factor W at different SoC.

Supplementary Table 1. EIS fitting results of the pristine Si/LPSCl composite based on the transmission line model.

Time (h)	$R_{e,\ bulk}$ (Ω cm^2)	$R_{e,\ int}$ (Ω cm^2)	R_{ion} (Ω cm^2)	Electronic conductivity (×10^{-6} S cm^{-1})	Ionic conductivity (×10^{-6} S cm^{-1})
0	100.0	4862.8	1794.8	9.2	25.3
1	198.8	5562.6	2057.6	7.9	22.1
2	143.9	5751.4	1961.8	7.7	23.2
3	172.1	5761.0	2061.2	7.7	22.0
4	146.2	5807.6	2061.1	7.6	22.0
5	140.4	5794.5	2147.9	7.7	21.2
6	170.3	5729.5	2173.9	7.7	20.9
7	159.1	5716.3	2088.2	7.7	21.8
8	152.0	5738.7	2084.4	7.7	21.8
9	174.8	5702.1	2217.6	7.7	20.5
10	179.5	5681.0	2228.9	7.8	20.4
11	158.3	5669.0	2253.0	7.8	20.2
12	214.7	5599.2	2109.9	7.8	19.5
13	173.3	5521.9	2358.5	8.0	19.3
14	169.7	5506.4	2396.1	8.0	19.0
15	129.9	5569.8	2318.1	8.0	19.6
16	161.7	5515.3	2387.4	8.0	19.0
17	137.1	5550.7	2353.2	8.0	19.3

Supplementary Table 2. EIS fitting results of the Si/LPSCl composite during resting at the open-circuit voltage after being discharged to E = –0.6 V, i.e., to E = 0.02 vs. Li^+/Li.

Time (h)	R_{SE} (Ω cm^2)	R_{comp} (Ω cm^2)	R_{int} (Ω cm^2)
1	10	25.0	26.2
2	10	25.1	29.4
3	10	25.4	33.0
4	10	25.6	35.7
5	10	25.8	37.6
6	10	26.2	39.5
7	10	26.6	41.7
8	10	26.8	43
9	10	27.1	44.6
10	10	27.4	46.2
11	10	27.7	47.9
12	10	27.8	49.5
13	10	27.9	51.2
14	10	28.2	53.2
15	10	28.3	54.3
16	10	28.5	57.1

Supplementary Table 3. Lattice parameters of unit cells of crystalline Li_xSi alloys (materials ID from Materials Project).[14]

Formula	Material ID	Crystal system	Lattice constant (Å)			Volume (Å^3)
			a	b	b	
$LiSi_3$	mp-975321	Tetragonal	3.75	3.75	8.05	113.16
LiSi	mp-570363	Tetragonal	9.33	9.33	5.74	499.42
$Li_{12}Si_7$	mp-1314	Orthorhombic	8.53	14.31	19.62	2396.02
Li_2Si	mp-27705	Trigonal	4.34	4.343	18.433	301.13
Li_7Si_3	mp-1201871	Trigonal	7.58	7.58	18.01	895.76
$Li_{13}Si_4$	mp-672287	Orthorhombic	4.42	7.90	15.01	524.13
Li_7Si_2	mp-27930	Orthorhombic	4.48	8.05	14.91	537.06
$Li_{15}Si_4$	mp-569849	Cubic	10.57	10.57	10.57	1179.61

Supplementary Table 4. Total Bader charge of Li atoms in amorphous structures along with the corresponding volume of different Li_xSi amorphous structures and electron concentration. Electron conductivity was obtained from BoltzTrap calculations.

Formula	Bader charge |e|	Volume (10^{-21} cm^3)	Electron concentration (10^{20} cm^{-3})	Li^+ ion concentration (10^{20} cm^{-3})	Electron conductivity (10^{-4} S/cm)
$LiSi_3$	51.37	3.62	1.42	1.77	1.48
LiSi	177.34	7.99	2.22	3.21	1.73
$Li_{12}Si_7$	155.25	4.79	3.24	4.01	3.42
Li_2Si	155.57	4.82	3.23	3.98	5.16
Li_7Si_3	135.36	3.58	3.78	3.49	5.28
$Li_{13}Si_4$	184.68	4.72	3.91	4.96	6.07
Li_7Si_2	194.52	4.83	4.02	5.22	6.38
$Li_{15}Si_4$	183.77	4.72	3.89	5.08	5.38

Supplementary Table 5. EIS fitting results of the SE-free Si anode during the relaxation of GITT.

Li_xSi	R_{SE} (Ω cm^2)	R_{bulk} (Ω cm^2)	R_{int} (Ω cm^2)
$Li_{0.094}Si$	10	64.9	44.9
$Li_{0.188}Si$	10	33.1	26.1
$Li_{0.281}Si$	10	17.2	20.1
$Li_{0.375}Si$	10	11.2	18.3
$Li_{0.469}Si$	10	8.5	14.7
$Li_{0.563}Si$	10	7.3	7.0
$Li_{0.656}Si$	10	7.0	11.3
$Li_{0.75}Si$	10	6.7	9.7
$Li_{0.844}Si$	10	6.6	9.6
$Li_{0.938}Si$	10	6.6	9.5
$Li_{1.031}Si$	10	6.6	10
$Li_{1.125}Si$	10	6.4	10.1
$Li_{1.219}Si$	10	6.4	10.1
$Li_{1.313}Si$	10	6.4	10.3
$Li_{1.406}Si$	10	6.2	10.4
$Li_{1.5}Si$	10	6.4	10.4
$Li_{1.594}Si$	10	6.4	10.6
$Li_{1.688}Si$	10	6.5	10.8
$Li_{1.781}Si$	10	6.6	11.0
$Li_{1.875}Si$	10	6.6	11.0
$Li_{1.969}Si$	10	6.7	11.1
$Li_{2.063}Si$	10	6.7	11.3
$Li_{2.156}Si$	10	6.9	11.1
$Li_{2.25}Si$	10	7	11.4
$Li_{2.344}Si$	10	7.1	11.6
$Li_{2.438}Si$	10	7.2	11.7
$Li_{2.531}Si$	10	7.2	11.9
$Li_{2.625}Si$	10	7.4	12.0
$Li_{2.719}Si$	10	7.6	12.0
$Li_{2.813}Si$	10	7.6	12.1
$Li_{2.906}Si$	10	7.7	12.0
Li_3Si	10	7.8	12.1
$Li_{3.094}Si$	10	7.8	12.2
$Li_{3.188}Si$	10	7.8	12.2
$Li_{3.281}Si$	10	7.9	12.3
$Li_{3.375}Si$	10	7.9	12.3
$Li_{3.469}Si$	10	7.9	12.2
$Li_{3.563}Si$	10	8.0	12.3
$Li_{3.656}Si$	10	8.0	12.3

Supplementary Table 6. EIS fitting results of the SE-free Si anode during resting at the open-circuit voltage after being discharged to E = –0.6 V, i.e., to E = 0.02 vs. Li^+/Li.

Time (h)	R_{SE} (Ω cm^2)	R_{bulk} (Ω cm^2)	R_{int} (Ω cm^2)
1	10	19.1	9.3
2	10	19.2	9.3
3	10	19.2	9.5
4	10	19.3	9.5
5	10	19.3	9.5
6	10	19.3	9.6
7	10	19.4	9.7
8	10	19.4	9.8
9	10	19.4	9.8
10	10	19.3	9.8
11	10	19.3	9.8
12	10	19.3	9.9
13	10	19.4	9.9
14	10	19.4	10
15	10	19.5	10.1
16	10	19.5	10.1
17	10	19.5	10.1
18	10	19.6	10.2
19	10	19.6	10.2
20	10	19.7	10.3

Supplementary Table 7. EIS fitting results of the SE-free Si anode after various cycles.

Cycle number	R_{SE} (Ω cm^2)	R_{bulk} (Ω cm^2)	R_{int} (Ω cm^2)
1	10	71.2	47.8
2	10	72.3	92.8
5	10	72.6	177.7
10	10	73.1	236.8

Supplementary Table 8 Parameters for the phase-field model

Parameter name	Value	Unit
Lithium diffusion coefficient D_{Li} in silicon	2.7×10^{-15}	m^2/s
Lithium ion diffusion coefficient D_{Li^+} in LPSCl	1.0×10^{-13}	m^2/s
Crack mobility L	1.0×10^{-5}	$\frac{m^3}{J \cdot s}$
Crack length scale l_0	0.03×10^{-6}	m
Youngs modulus of silicon E_{Si}	120,20	GPa
Youngs modulus of LPSCl	25	GPa
Poisson ratio ν silicon and LPSCl	0.3	-
Yield stress and hardening moduli of silicon	1.0, 2.5	GPa
Yield stress and hardening moduli of LPSCl	0.3, 1.5	GPa
C-rate	5	-

References

1 Perdew, J. P., Burke, K. & Ernzerhof, M. Generalized gradient approximation made simple. *Phys. Rev. Lett.* **77**, 3865 (1996).

2 Momma, K. & Izumi, F. VESTA: a three-dimensional visualization system for electronic and structural analysis. *J. Appl. Crystallogr.* **41**, 653-658 (2008).

3 Adeli, P. *et al.* Boosting solid-state diffusivity and conductivity in lithium superionic argyrodites by halide substitution. *Angew. Chem. Int. Ed.* **58**, 8681-8686 (2019).

4 Alvarado, J. *et al.* Bisalt ether electrolytes: a pathway towards lithium metal batteries with Ni-rich cathodes. *Energy Environ. Sci.* **12**, 780-794 (2019).

5 Li, W., Asl, H. Y., Xie, Q. & Manthiram, A. Collapse of $LiNi_{1-x-y}Co_xMn_yO_2$ lattice at deep charge irrespective of nickel content in lithium-ion batteries. *J. Am. Chem. Soc.* **141**, 5097-5101 (2019).

6 Xu, X. *et al.* Kinetically stabilized ferroelectricity in bulk single-crystalline HfO_2: Y. *Nat. Mater.* **20**, 826-832 (2021).

7 Okhotnikov, K., Charpentier, T. & Cadars, S. Supercell program: a combinatorial structure-generation approach for the local-level modeling of atomic substitutions and partial occupancies in crystals. *J. cheminformatics* **8**, 1-15 (2016).

8 Jiang, M. *et al.* Se-doped Li_6PSCl and $Li_{5.5}PS_{4.5}Cl_{1.5}$ with improved ionic conductivity and interfacial compatibility: a high-throughput DFT study. *J. Mater. Chem. C* **10**, 18294-18302 (2022).

9 Zhao, X., Ceresoli, D. & Vanderbilt, D. Structural, electronic, and dielectric properties of amorphous ZrO_2 from ab initio molecular dynamics. *Phys. Rev. B* **71**, 085107 (2005).

10 He, X., Zhu, Y., Epstein, A. & Mo, Y. Statistical variances of diffusional properties from ab initio molecular dynamics simulations. *npj Computational Mater.* **4**, 18 (2018).

11 Hoover, W. G. Canonical dynamics: Equilibrium phase-space distributions. *Phys. Rev. A* **31**, 1695 (1985).

12 Nosé, S. A unified formulation of the constant temperature molecular dynamics methods. *J. Chem. Phys.* **81**, 511-519 (1984).

13 Wang, L., Ding, B. & Guo, Y. Spin-polarized transport behavior induced by asymmetric edge hydrogenation in hybridized zigzag boron nitride and graphene nanoribbons. *J. Electron. Mater.* **48**, 321-328 (2019).

14 Jain, A. *et al.* Commentary: The Materials Project: A materials genome approach to accelerating materials innovation. *APL materials* **1**, 011002 (2013).

15 Steinbach, I. *et al.* A phase field concept for multiphase systems. *Physica D: Nonlinear Phenomena* **94**, 135-147 (1996).

16 Chen, L.-Q. Phase-field models for microstructure evolution. *Annual review of materials research* **32**, 113-140 (2002).

17 Svendsen, B., Shanthraj, P. & Raabe, D. Finite-deformation phase-field chemomechanics for multiphase, multicomponent solids. *J. Mech. Phys. Solids* **112**, 619-636 (2018).

18 Guyer, J. E., Boettinger, W. J., Warren, J. A. & McFadden, G. B. Phase field modeling of electrochemistry. I. Equilibrium. *Phys. Rev. E* **69**, 021603 (2004).

19 Liang, L. & Chen, L.-Q. Nonlinear phase field model for electrodeposition in electrochemical systems. *Appl. Phys. Lett.* **105**, 263903 (2014).

20 Chen, L. *et al.* Modulation of dendritic patterns during electrodeposition: A nonlinear phase-field model. *J. Power Sources* **300**, 376-385 (2015).

21 Miehe, C., Hofacker, M. & Welschinger, F. A phase field model for rate-independent crack propagation: Robust algorithmic implementation based on operator splits. *Computer Methods in Applied Mechanics and Engineering* **199**, 2765-2778 (2010).

22 Simo, J. C. & Hughes, T. J. *Computational inelasticity*. Vol. 7 (Springer Science & Business Media, 2006).

23 Weppner, W. & Huggins, R. Electrochemical investigation of the chemical diffusion, partial ionic conductivities, and other kinetic parameters in Li_3Sb and Li_3Bi. *J. Solid State Chem.* **22**, 297-308 (1977).
